# Modelling the Incidence of *Plasmodium vivax* and *Plasmodium falciparum* Malaria in Afghanistan 2006–2009

**DOI:** 10.1371/journal.pone.0102304

**Published:** 2014-07-17

**Authors:** Victor A. Alegana, Jim A. Wright, Sami M. Nahzat, Waqar Butt, Amad W. Sediqi, Naeem Habib, Robert W. Snow, Peter M. Atkinson, Abdisalan M. Noor

**Affiliations:** 1 Spatial Health Metrics Group, Department of Public Health, KEMRI-Wellcome Trust, Nairobi, Kenya; 2 Centre for Geographical Health Research, Geography and Environment, University of Southampton, Highfield Southampton, United Kingdom; 3 National Malaria and Leishmaniasis Control Programme, Ministry of Public Health, Kabul, Afghanistan; 4 Malaria and Leishmaniasis, WHO Office, Kabul, Afghanistan; 5 Centre for Tropical Medicine, Nuffield Department of Clinical Medicine, University of Oxford, Oxford, United Kingdom; Centro de Pesquisa Rene Rachou/Fundação Oswaldo Cruz (Fiocruz-Minas), Brazil

## Abstract

**Background:**

Identifying areas that support high malaria risks and where populations lack access to health care is central to reducing the burden in Afghanistan. This study investigated the incidence of *Plasmodium vivax* and *Plasmodium falciparum* using routine data to help focus malaria interventions.

**Methods:**

To estimate incidence, the study modelled utilisation of the public health sector using fever treatment data from the 2012 national Malaria Indicator Survey. A probabilistic measure of attendance was applied to population density metrics to define the proportion of the population within catchment of a public health facility. Malaria data were used in a Bayesian spatio-temporal conditional-autoregressive model with ecological or environmental covariates, to examine the spatial and temporal variation of incidence.

**Findings:**

From the analysis of healthcare utilisation, over 80% of the population was within 2 hours’ travel of the nearest public health facility, while 64.4% were within 30 minutes’ travel. The mean incidence of *P. vivax* in 2009 was 5.4 (95% Crl 3.2–9.2) cases per 1000 population compared to 1.2 (95% Crl 0.4–2.9) cases per 1000 population for *P. falciparum*. *P. vivax* peaked in August while *P. falciparum* peaked in November. 32% of the estimated 30.5 million people lived in regions where annual incidence was at least 1 case per 1,000 population of *P. vivax;* 23.7% of the population lived in areas where annual *P. falciparum* case incidence was at least 1 per 1000.

**Conclusion:**

This study showed how routine data can be combined with household survey data to model malaria incidence. The incidence of both *P. vivax* and *P. falciparum* in Afghanistan remain low but the co-distribution of both parasites and the lag in their peak season provides challenges to malaria control in Afghanistan. Future improved case definition to determine levels of imported risks may be useful for the elimination ambitions in Afghanistan.

## Background

Since the Soviet invasion in 1979, Afghanistan has experienced prolonged periods of insecurity and political instability. Consequently it has some of the poorest socio-economic and health status indicators globally. The country is ranked the thirteen lowest on the human development index [1] and has a child mortality rate of 97 deaths before the age of five years for every 1000 children born [2], [3]. In Afghanistan, malaria is an important disease with approximately half the population at risk [4], [5], [6].

Malaria transmission in the country is constrained by altitude, the rugged topography, patchy rainfall and extreme aridity [7]. There is no active malaria transmission in areas greater than 2000 metres above mean sea level [8], while transmission is unstable in areas with limited annual rainfall. There are at least six malaria vectors in Afghanistan namely: the *Anopheles superpictus*, *An. culicifacies*, *An. hycranus*, *An. Pulcherimus, An. fluviatilis, and An. stephensi*. The latter two are mainly found in the eastern provinces [4], [9]. Malaria infections are predominantly due to the *Plasmodium vivax* parasite although *Plasmodium falciparum* infections exist [8].

Afghanistan has a long history of malaria control dating back to the formation of the Directorate General of Preventive Medicine and Primary Health Care in 1948 [7]. Earlier vector control efforts focused on spraying using dichlorodiphenyltrichloroethane (DDT) and by 1970 the *An. superpictus* was almost eradicated [7], [10], [11]. After the Soviet invasion, the national program gradually weakened and had almost ceased to function [7], [12]. Chloroquine resistance and population movement, mainly from returning refugees, contributed to an increase in malaria burden in Afghanistan [12], [13], [14]. Since 2000, however, substantial resources have been invested in malaria control in Afghanistan with support from the Global Fund to fight AIDS, Tuberculosis and Malaria, the United States Agency for International Development (USAID) as well as other agencies [15]. Despite the insecurity and infrastructure challenges, progress has been made in reducing the burden [5]. A recent malaria indicator survey (MIS) conducted in 2011 showed an average prevalence of less than 1% for both *P. vivax* and *P. falciparum* nationally while 76% of household clusters had no residents infected.

In the national malaria strategy of 2008–2013, Afghanistan aimed to reduce, by 60%, the malaria morbidity by 2013 and reduce *P. falciparum* cases to near zero with the aim of eventually interrupting its transmission [15]. The main interventions were coverage with vector control, parasitological diagnosis and treatment with effective antimalarials. In addition, a cross-border initiative was launched with Tajikistan to reduce the risk of imported infections to Tajikistan and to eliminate *P. falciparum* malaria in three border districts.

To track progress towards the national targets, the National Malaria and Leishmaniasis Control Programme (NMLCP) and partners established a routine information system to report monthly malaria cases by health facility [16]. The system, however, captured passively detected case data from only the public health system and contained both clinically diagnosed and parasitologically confirmed *P. vivax* and *P. falciparum* cases. Passive case detection, usually from HMIS, is hindered by the challenges of the low parasite confirmation rates which inflate reported malaria caseloads. In addition, low reporting rates tend to underestimate disease burdens because of the spatially and temporally incomplete data [17]. To provide more reliable estimates of disease burden, techniques are required that can adjust for these deficiencies by smoothing crude incidence rates; filling in gaps where no health reports have been assembled; and adjusting for the rate of facility utilisation since only a proportion of actual cases present at a facility [18].

In this study, a formal spatial and temporal approach, that incorporates a variety of data sources to estimate malaria incidence by district from 2006–2009 in Afghanistan, was developed. First, nationally representative household survey data from the 2011 MIS were used to characterize the utilisation of public health facilities and subsequently develop the denominator (catchment population) weighted by probability of health facility use for fever treatment. Secondly, malaria cases reported at the health facilities were used to model incidence of *P. vivax* and *P. falciparum* spatially and temporally using a Bayesian approach [19]. The clinically reported cases were adjusted using species-specific slide positivity rates observed at the facility and combined with parasite species confirmed cases to calculate the numerator. Slide positivity is the ratio of the number of positive malaria cases to the total number of people examined usually expressed as a percentage (rate). The combination of the adjusted cases and catchment populations were then used to compute the incidence of both *P. vivax* and *P. falciparum*.

## Methods

### Health management information structure in Afghanistan

Afghanistan is divided into 34 administrative provinces. Healthcare is delivered mainly through the Basic Package for Health Services (BPHS) and the Essential Package for Hospital Services (EPHS) constituted in 2002 by the Ministry of Public Health (MoPH) [20], [21], [22]. In a bid to increase coverage, the BPHS was expanded through the contracting out of services to NGOs and MoPH partners [21], [23]. The BHC constitutes clinics, health posts and Maternal Child Health (MCH) centres and Comprehensive Health Centres (CHC). This is linked to EPHS made up of the District Hospitals (DH) (first referral level) and regional or provincial (tertiary) hospitals. At village level community health workers manage the health posts and treat mild conditions and, in some cases, Mobile Health Teams (MHTs) are used [20], [24]. In terms of data reports, tally sheets are filled at these lower-tier facilities and aggregated at the next tier facilities (CHC) which are then forwarded to regional directorates [16]. Thus, the health posts serve as a support network for the health centres and sometimes malaria cases are reported at the health centre rather than the individual health unit. The basic health centres link the basic service providers at the community level with the next service tier (the CHC) that are, in turn, linked to district hospitals and regional referral hospitals. Thus, where no regional or tertiary facility exists, district hospitals are the main referral centres. HMIS reports are also compiled the regional level and distributed to the national management level. Inpatient facilities are provided mainly at the tertiary level [20]. Parasitological diagnosis is conducted at higher tier facilities (Hospitals) where laboratory facilities exist while clinical diagnosis is predominantly used at health posts. The 2010 national malaria treatment guidelines outline the scale up of diagnostics at all health facilities to ensure diagnosis prior to treatment.

### Data

The malaria case data were obtained from HMIS through the Afghanistan National Malaria and Leishmaniasis Control Programme (NMLCP). This consisted of records from 1,629 public health facilities for a 48-month period from 2006 to 2009. Data represented aggregate monthly cases of *P. falciparum* and *P. vivax*. Of the 1,629 health facilities, 1,587 had reported malaria cases based on both clinical and parasitology examination. Parasitological diagnosis (microscopy or RDTs) was conducted at higher-tier facilities (hospitals and health centres) where laboratory facilities exist while clinical diagnosis was predominantly used at lower-level facilities such as health posts (File S1). No cases were examined or reported for 228 facilities which were treated as missing data while data for mobile units (*n = 93*) were omitted from the final analysis since they serve as outreach centres from major facilities. The missing spatial and temporal structures of data were imputed as ‘*NAs*’ and predictions made at missing locations. The spatial coordinates of health facilities were obtained from the Afghan Management Information Systems (AMIS) (http://www.aims.org.af/), which was formerly managed by the United Nations Office for the Coordination of Humanitarian Affairs (UNOCHA) and the United Nations Development Programme (UNDP) in the early 2000s, but became a national independent Non-Governmental Organisation (NGO) in 2008. These facilities were either mapped using non-differential handheld global positioning systems (GPS) receivers during the assessment surveys or in some cases the longitude and latitude were established using a village or settlement database. For analysis, the facilities were classified into three broad categories that combined: basic facilities made up of health posts (HPs), clinics and maternal health centres (MCH); health centres; and hospitals.

Data for modelling health care utilisation for treatment of fever was obtained from the national MIS carried out between September and October 2011 (*n* = 15,442 individuals)[25]. The MIS was conducted in 21 provinces, across the diverse malaria strata (medium to high risks; low risk; and very low or potentially malaria free areas) in Afghanistan, but excluded the southern regions for security reasons. A multi-stage probability sampling design was adopted in line with other MIS surveys conducted in sub-Saharan countries [26]. At the first stage clusters or villages were selected randomly in a district via probability sampling while at the second stage, households within the selected clusters were sampled randomly [25]. Self-reported treatment seeking behaviour, disaggregated by healthcare sector, was recorded for all household members that reported an episode of fever two weeks prior to the survey. A gridded population surface for Afghanistan was obtained from Asiapop at 100 m x 100 m spatial resolution (http://www.worldpop.org.uk/)[27].

## Analysis

### Analysis of public sector utilisation and defining the denominator for modelling incidence

A combination of land cover, elevation, road and river data layers was used to generate a gridded cost surface of travel time between patient origins (households) and destinations (public health facilities) as described elsewhere [28] and in File S1. Travel times were extracted for each MIS cluster and used to predict the probability of health facility attendance based on reported fever treatment. A probability of attendance was modelled spatially at 1 km by 1 km resolution and combined with population density to generate a population-weighted surface for fever treatment. The population-weighted counts, used in modelling incidence, were extracted based on a 2-hour cut off based on the modelled distance decay curves (SI). The catchment population was adjusted for reporting rates at the facilities calculated as a ratio of received reports to the expected number over the four-year period.

### Modelling incidence of *P. falciparum* and *P. vivax* in Afghanistan

To model the incidence of malaria, HMIS data were compiled from cases aggregated at each facility for each month. A number of environmental covariates such temperature suitability index (TSI), precipitation and enhanced vegetation index (EVI) that are known to affect malaria transmission were assembled (SI). The selection of covariates was based on previous studies [19] as well as aiming for a minimum set to achieve parsimony based on *bestglm* package in R [29]. These covariates were extracted and matched to each data point in space and time. Environmental covariates were used in a Bayesian zero-inflated conditional autoregressive (CAR) model to predict incidence at the district level. Since 60% of data were zeros, a zero – inflated Poisson distribution was used, generalized as [30], [31];
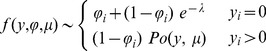
(1)for the 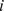 ^th^ space-time observation and 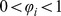. The probability is defined via a two-component mixed model such that the probability is 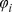 with ‘structural’ zero or defaulting to a general Poisson model (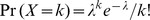). In general 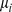 can depend on a set of covariates such that: 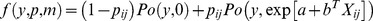
(2)with 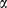 (equation 2) forming the intercept modified by a 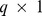 vector of 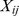
covariates with unknown coefficients 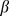. Further, 
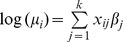
 while 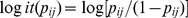. Thus, the zero-inflated probability increases the chance of predicting ‘structural’ zero [32], [33]. Random effects were introduced at three levels of the health facility, district and province in the Bayesian framework. A spatial effect prior 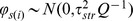 was introduced at the district level to account for spatial heterogeneity. Model specification was completed by assigning priors to the remaining hyper-parameters (the unstructured random effects). Inverse Gamma priors 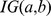 were assigned to precision hyperparameters for these unstructured effects components 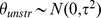. The time interaction was modelled as a first-order auto-regressive process, 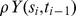 with the first term coming from a stationary distribution 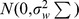 that depends on past values for 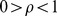 [34]. Full details of implementation can be found in File S1.

Posterior predictions were made at the district level along with associated standard errors. Four spatio-temporal models were compared to assess the effect of the introduced random effects at province and facility level as well as the inclusion of the covariates. The first two models (referred to as M1 and M2) did not include any covariates with random effects excluded for the first model (M1). The other two models (M3 and M4) included environmental covariates, with M3 excluding random effects at facility and province level. Comparisons were made using the deviance information criterion (DIC) [35]. This approach simplifies model selection to a single value, which can be easily tabulated for comparison with proper Bayesian interpretation. A subset comprising 10% of the data selected randomly was used independently to compare posterior prediction against the crude incidence. Additionally, model checking was implemented by assessing the variance and the standard error of the predictive distribution [36].

## Results

### Data characteristics and public sector utilisation in Afghanistan

In modelling healthcare utilisation, a list of the universe of public health facilities was used (*n* = 1,581) from the 34 provinces. There were more health posts (*n* = 754) compared to health centres (*n* = 698) and hospital (*n* = 129). The majority were run by NGOs that work in partnership with the Ministry of Public Health (MOPH). The malaria case reporting rate was low for basic health facilities (an average of 33% for the four years) compared to hospitals and health centres where the reporting rate was >70%. Of the estimated population (32.3 million) in 2011, 27.8 million (85.8%) were estimated to be within 2 hours’ of travel of a public health facility; 17.9 million (64.4%) were within 30 minutes. Approximately 13.1 million (47.4%) were within distances where the probability of attendance was ≥60% (SI).

### Posterior predictions of incidence of *P. falciparum* and *P. vivax*

Table 1 lists the four Bayesian spatio-temporal models implemented along with associated model parameters for both *P. vivax* and *P. falciparum*. According to the DIC, the fourth model (M4) provided the best trade-off between model fit and parsimony compared to the other three models, although with more effective parameters 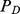. For both *P. vivax* and *P. falciparum*, the standard error in M4 of the predictive distribution was also lower. This model was subsequently selected for analysis of incidence of *P. vivax* and *P. falciparum*. Overall mean error of the crude incidence and the predicted incidence per 1000 population per year, based on a 10% validation set was −0.30 and −0.44 for *P. vivax* and *P. falciparum* respectively showing an overall tendency to under-predict by less than 0.5 incident cases per 1000 population. The Pearson correlation based on the validation set was 0.63 for *P. vivax* and 0.62 for *P. falciparum*. Table 2 lists the posterior summaries of the parameters representing the fixed effects, the unstructured components, and the temporal and spatial parameters for both the *P. vivax* and *P. falciparum* models. None of the covariate parameters were significant at 95% Bayesian credible interval (Crl) based on the *P. falciparum* model but temperature suitability (0.123, 95% Crl 0.046–0.202) was significant based on the *P. vivax* model. All other model parameters were significant at 95% Crl.

**Table 1:** Comparison of four Bayesian models with and without random effects and covariates (M1 with no random effects or environmental covariates; M2: with random effects but no environmental covariates; M3: with environmental covariates but no random effects; M4 with random effects and environmental covariates (Rainfall, TSI and EVI).

	Model	DIC	P_D_	Mlik(Integration)	Variance ofpredictivedistribution	Std error ofpredictivedistribution	Mean Error	R*^2^*
*P. falciparum*	M1	3670.00	86.80	−1824.57	0.002	1.026	-	-
	M2	3596.90	95.60	−1824.94	0.005	1.042	-	-
	M3	3599.48	90.64	−1821.78	0.002	1.026	-	-
	M4	3570.76	96.85	−1804.94	0.002	1.022	−0.442	0.619
*P. vivax*	M1	20933.49	203.48	−10571.74	0.001	1.054	-	-
	M2	20781.31	301.97	−10538.10	0.001	1.049	-	-
	M3	20935.46	206.49	−10593.93	0.001	1.052	-	-
	M4	20780.64	301.46	−10554.87	0.001	1.047	−0.308	0.629

**DIC**: Deviance information Criterion, **P_D_**: Effective number of parameters, **Mlik**: Maximum likelihood estimate.

**Table 2:** Parameters of the selected Bayesian models (M4) for both *P. falciparum* and *P. vivax* (sequentially as intercept β_0_, EVI, TSI, Precipitation, random effects at (facility, district and province), temporal parameter and spatial CAR prior effect φ, SD is the Standard Deviation).

	Parameter	Mean	SD	5%	50%	95%
*P. falciparum*	Intercept (β_0_)	−3.630	0.387	−4.244	−3.633	−3.008
	EVI (β_1_)	−0.031	0.079	−0.162	−0.031	0.099
	TSI (β_2_)	0.164	0.127	−0.042	0.163	0.334
	Precipitation (β_3_)	0.008	0.051	−0.077	0.008	0.091
	Facility random effect (τ_1_)	1.940	1.903	0.192	1.380	5.534
	District random effect (τ_2_)	2.484	0.829	1.355	2.369	4.010
	Province random effect (τ_3_)	3.668	1.164	2.040	3.521	5.838
	Rho for the month (ρ)	0.849	0.117	0.617	0.881	0.969
	Spatial effect (φ)	5.492	4.535	0.698	2.376	20.970
*P. vivax*	Intercept (β_0_)	−2.065	0.240	−2.451	−2.069	−1.662
	EVI (β_1_)	−0.026	0.019	−0.058	−0.026	0.005
	TSI (β_2_)	0.124	0.048	0.046	0.124	0.202
	Precipitation (β_3_)	0.013	0.011	−0.005	0.013	0.031
	Facility random effect (τ_1_)	8.383	1.778	6.095	8.057	11.750
	District random effect (τ_2_)	2.081	1.976	0.181	1.500	5.888
	Province random effect (τ_3_)	7.972	3.953	3.897	6.922	15.530
	Rho for the month (ρ)	0.728	0.098	0.551	0.737	0.872
	Spatial effect (φ)	3.141	0.983	1.759	3.024	4.933

The betas represent the fixed effects of the covariates.

**Figure 1:**
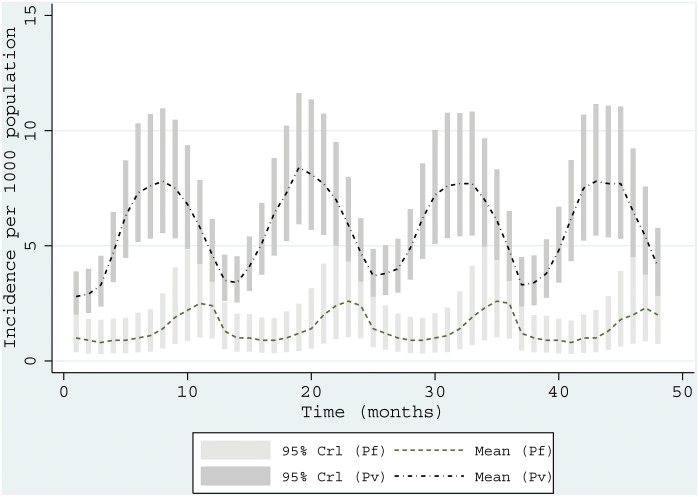
Time series of two malaria parasites. Plots showing the predicted monthly (*n* = 48 months) incidence for (2006–2009) for *P. vivax* (mean as top dash-dot line) and for *P. falciparum* (mean as green dash line) with error bars for each moth showing 95% Bayesian credible interval (Crl). *P. vivax* formed the most burden in Afghanistan and its incidence peaked in July and August compared to *P. falciparum* that peaked later in the year in November.

**Figure 2:**
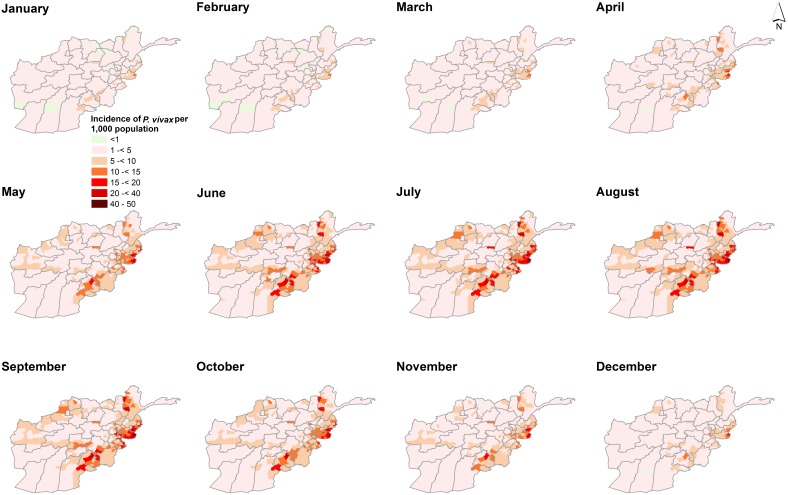
Monthly maps of *P. vivax*. Maps showing the predicted posterior mean monthly incidence of *P. vivax* per 1000 population for Afghanistan in 2009 using a Bayesian CAR model with environmental covariates (rainfall, TSI and EVI). Cases comprised of parasitologically confirmed and clinical cases corrected for slide positivity rates at the facility for a four-year period (2006–2009). Random unstructured effects were included at the facility level to account for regional heterogeneity. The highest burden of *P. vivax* (exceeding 15 cases per 1000 population) was in southeastern and the eastern regions bordering Pakistan.

**Figure 3:**
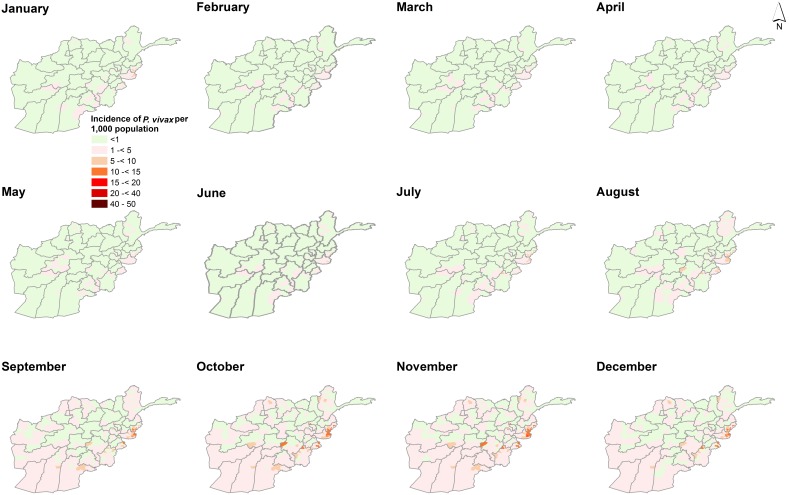
Monthly maps of *P. falciparum*. Maps showing the predicted monthly incidence of *P. falciparum* per 1000 population for Afghanistan in 2009 using a Bayesian CAR model with environmental covariates (rainfall, TSI and EVI). Malaria cases comprised of parasitologically confirmed and clinical cases corrected for slide positivity rates at the facility. Random unstructured effects were included at the facility level to account for regional heterogeneity. *P. falciparum* constitutes less than 10% of the malaria burden in Afghanistan and experienced a late peak in the year (November).

**Figure 4:**
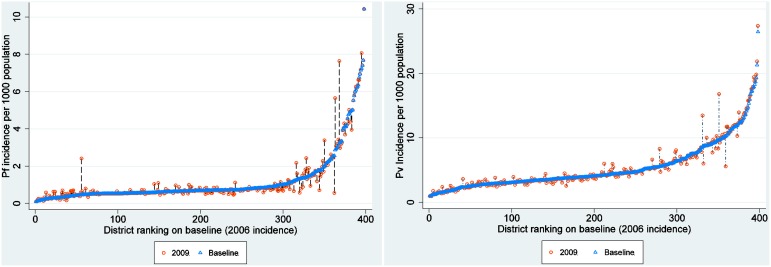
Incidence change plot at district level. Plot showing the differences in malaria incidence per 1000 population (*y*-axis) between the baseline year (2006) plotted as blue triangles and incidence for 2009 (hollow red circles). The *x*-axis represents districts (*n* = 398). The positive change denoting increase in plotted vertically upwards from the baseline year while negative denoting a reduction in incidence is vertically downwards from the baseline with the length indicating magnitude of change. Overall percentage change for *P. vivax* was 3.0 and 5.9 for *P. falciparum*.

Figure 1 shows the monthly variation of incidence for *P. vivax* and *P. falciparum* for the four-year period. The incidence of *P. vivax* peaked in August (7.611 95% Crl 4.849–11.721) compared to *P. falciparum* which peaked in November (mean incidence per 1,000 population was 2.403 95% Crl 0.929–5.276). *P. falciparum* was lowest in May (0.830 95% Crl 0.303–1.783). Figure 2 and Figure 3 shows maps of monthly incidence of *P. vivax* and *P. falciparum*, respectively, at district level. The incidence of *P. falciparum* was generally very low compared to *P. vivax*. Nangahar, Kabul and Kunar had highest estimated clinical burden of *P. vivax* and *P. falciparum* while lowest estimated burden was in districts bordering Iran in Nimroz and Farah and in northern Afghanistan. The predicted mean incidence in the most recent data year (2009) for *P. vivax* was 5.4 (95% Crl 3.2–9.2) cases per 1,000 population and 1.2 (95% Crl 0.4–2.9) cases per 1,000 population for *P. falciparum*. Comparison between the baseline in 2006 and in 2009 showed small change in incidence (4.9, 95% Crl 3.0–7.8 and 5.1, 95% Crl 3.2–8.1 respectively for *P. vivax*; 1.1, 95% Crl 0.3–2.4 and 1.1, 95% Crl 0.3–2.5 respectively for *P. falciparum*) (Figure 4). However, there was a slight increase in malaria incidence in 2008 for both *P. vivax* and *P. falciparum* as predicted by the model, but, subsequently dropped to the 2006 level in 2009. The mean percentage change in incidence in the 34 provinces between the baseline year and 2009 for *P. vivax* was 3.0 and 5.9 for *P. falciparum* (Table 3). *P. vivax* reduced in 17 of the 34 provinces in Afghanistan while *P. falciparum* reduced in 13 provinces.

**Table 3:** Predicted mean malaria incidence per 1000 population for 2009 and estimated population at risk by Province for *P. vivax and P. falciparum*.

	*Plasmodium vivax* incidence per 1,000 population									*Plasmodium falciparum* incidence per 1,000 population								
Province	Estimated Pv Clinical burden	Mean incidence 2009	sd		Percentage change Baseline (2006 and 2009	<1 (%)	1 -<5 (%)	5 -<10 (%)	≥10 (%)	Estimated Pf Clinical burden	Mean incidence 2009	sd	Percentage change Baseline (2006 and 2009	<1 (%)	1 -<5 (%)	5 -<10 (%)		Total Population
Kabul	19,788	4.3	1.1		0.59	0	4,513,039 (98.1)	72,584 (1.6)	16222 (0.4)	5,016	1.1	0.7	-0.52	4,132,170 (89.8)	425,758 (9.3)	43,917 (1)		4,601,845
Kapisa	2,497	5.4	0.8		0.59	0	215,825 (46.8)	245,789 (53.2)	0	268	0.6	0.5	-0.21	461,613 (100)	0	0		461,613
Logar	1,988	4.4	1.1		-2.01	0	308,882 (68.2)	143,913 (31.8)	0	317	0.7	0.6	-5.99	425,918 (94.1)	26,877 (5.9)	0		452,795
Panjshir	777	5.5	1.5		1.24	0	0	123,418 (86.7)	18879 (13.3)	203	1.4	0.8	1.74	99,628 (70.0)	23,790 (16.7)	18,879 (13.3)		142,298
Parwan	2,806	3.4	0.9		-4.04	0	778,082 (93.2)	57,015 (6.8)	0	576	0.7	0.6	-3.74	778,082 (93.2)	57,015 (6.8)	0		835,096
Wardak	2,439	3.8	1.1		-1.13	0	515,633 (79.9)	129,611 (20.1)	0	400	0.6	0.5	-3.68	573,533 (88.9)	71,711 (11.1)	0		645,244
Bamyan	2,172	4.3	1.3		-4.79	0	465,596 (92.2)	0	39633 (7.8)	273	0.5	0.5	-8.76	505,228 (100)	0	0		505,228
Day Kundi	2,280	4.2	1.5		0.19	0	143,333 (26.2)	403,394 (73.8)	0	306	0.6	0.6	-0.02	546,727 (100)	0	0		546,727
Kunar	6,897	13.3	2.0		2.61	0	0	0	517,421 (100)	2,013	3.9	1.3	-0.03	175,647 (33.9)	182,013 (35.2)	159,762 (30.9)		517,421
Laghman	4,114	8.1	0.6		2.91	0	48,935 (9.7)	456,482 (90.3)	0	510	1.0	0.5	1.68	386,431 (76.5)	118,986 (23.5)	0		505,417
Nangarhar	23,043	13.3	0.9		5.48	0	0	733,865 (42.5)	993,460 (57.5)	6,391	3.7	1.2	3.94	0	1,453,897 (84.2)	273,428 (15.8)		1,727,324
Nuristan	1,043	5.9	1.1		3.20	0	35,830 (20.3)	112,523 (63.6)	28511 (16.1)	108	0.6	0.5	2.67	176,863 (100)	0	0		176,863
Badakhshan	6,895	5.4	1.4		0.59	0	247,984 (19.4)	785,904 (61.5)	243,005 (19)	1,302	1.0	0.8	0.93	618,838 (48.5)	658,054 (51.5)	0		1,276,892
Baghlan	3,039	2.9	1.0		2.05	0	1,040,766 (100)	0	0	385	0.4	0.4	5.79	1,040,766 (100)	0	0		1,040,766
Kunduz	4,639	4.1	0.7		-0.82	0	755,118 (66.4)	381,799 (33.6)	0	432	0.4	0.4	2.59	1,136,917 (100)	0	0		1,136,917
Takhar	3,452	3.1	0.7		1.73	0	1,128,142 (100)	0	0	463	0.4	0.3	3.65	1,128,142 (100)	0	0		1,128,142
Balkh	4,091	2.9	1.1		10.56	408,202 (28.9)	1,002,618 (71.1)	0	0	818	0.6	0.5	15.39	1,410,820 (100)	0	0		1,410,820
Faryab	5,209	4.8	1.3		-0.05	0	498,575 (45.8)	591,181 (54.2)	0	708	0.7	0.5	0.25	971,677 (89.2)	118,079 (10.8)	0		1,089,756
Jawzjan	2,583	4.1	1.3		-2.39	0	521,918 (83)	106,563 (17)	0	760	1.2	0.9	8.69	255,757 (40.7)	372,724 (59.3)	0		628,480
Samangan	1,219	2.8	1.0		-1.86	0	441,833 (100)	0	0	239	0.5	0.5	-4.16	441,833 (100)	0	0		441,833
Sari Pul	2,548	3.7	1.0		3.69	0	604,485 (88.5)	78,646 (11.5)	0	266	0.4	0.4	20.25	683,132 (100)	0	0		683,132
Khost	5,613	8.5	1.3		10.31	0	57,439 (8.7)	343,942 (52.2)	257,366 (39.1)	1,719	2.6	1.1	17.70	13,623 (2.1)	426,758 (64.8)	218,366 (33.1)		658,747
Paktika	3,037	6.0	1.9		-4.60	0	25,624 (5.0)	453,431 (89.1)	29,674 (5.8)	829	1.6	1.0	-13.29	171,714 (33.8)	216,884 (42.6)	120,131 (23.6)		508,729
Paktya	4,458	6.9	1.1		-0.44	0	115,091 (17.8)	432,258 (67)	97,766 (15.2)	387	0.6	0.5	1.12	645,114 (100)	0	0		645,114
Ghazni	9,033	6.3	1.2		2.46	0	530,263 (36.7)	739,121 (51.2)	173,664 (12)	2,165	1.5	1.0	16.31	321,390 (22.3)	1,084,947 (75.2)	36,711 (2.5)		1,443,048
Hilmand	2,859	2.8	1.1		-0.26	0	1,039,697 (100)	0	0	1,071	1.0	0.8	1.90	930,158 (89.5)	109,539 (10.5)	0		1,039,697
Kandahar	5,644	4.1	1.2		-0.08	0	1,161,551 (84.2)	178,308 (12.9)	40,003 (2.9)	1,421	1.0	0.8	-4.06	1,130,774 (81.9)	249,088 (18.1)	0		1,379,862
Nimroz	577	3.1	1.2		-2.38	0	187,872 (100)	0	0	178	1.0	0.8	-7.58	86,898 (46.3)	100,974 (53.7)	0		187,872
Uruzgan	1,601	4.0	1.1		-0.88	0	306,799 (75.9)	97,595 (24.1)	0	311	0.8	0.7	0.87	332,451 (82.2)	71,944 (17.8)	0		404,395
Zabul	2,621	7.5	1.6		-6.88	0	20,586 (5.9)	144,845 (41.3)	185,415 (52.8)	379	1.1	0.8	-9.01	149,910 (42.7)	200,936 (57.3)	0		350,846
Badghis	2,257	4.1	0.9		1.92	0	534,183 (96.8)	17,642 (3.2)	0	353	0.6	0.5	7.60	551,825 (100)	0	0		551,825
Farah	1,459	2.5	1.0		-3.19	0	581,449 (100)	0	0	494	0.9	0.8	0.59	484,949 (83.4)	96,500 (16.6)	0		581,449
Ghor	3,141	4.1	1.4		-1.07	0	468,809 (60.9)	300,924 (39.1)	0	708	0.9	0.9	2.08	459,819 (59.7)	309,915 (40.3)	0		769,733
Hirat	7,532	3.6	1.2		16.37	0	2,098,175 (100)	0	0	1,133	0.5	0.5	23.14	209,8175 (100)	0	0		2,098,175
	**165,712**	**5.4**	**1.2**		**1.62**	**408,202 (1.3)**	**20,394,129 (66.7)**	**7,130,753 (23.3)**	**2,641,017 (8.6)**	**36,077**	**1.2**	**0.7**	**2.26**	**23,326,522 (76.3)**	**6,376,386 (20.9)**	**871,194 (2.8)**		**30,574,102**

**SD: Standard Deviation.**

Table 3 provides summaries of population at risk by region. Of the 30.6 million people in 2009, the estimated burden of *P. vivax* in 2009 was 165,712 compared to 36,077 for *P. falciparum*. Approximately 32% of the population lived in regions where *P. vivax* was greater than 1 case per 1000 population compared to 23.7% for *P. falciparum*. About 1.3% of the population were estimated to live in districts with <1 case per 1,000 population and the majority (66.7%) in districts of 1 to<5 *P. vivax* cases per 1,000 population. Of the remaining population, only 23.3% were in districts with 5 to<10 cases per 1,000 population, 8.4% in 10 to<20 cases per 1,000 population and 0.3% of the population. The latter comprised of populations in eastern Afghanistan in Kunar and Nangarhar provinces. For *P. falciparum* case incidence, 76.3% of the population lived in districts where annual incidence was <1 per 1,000 population, while 20.9% lived in areas were incidence of *P. falciparum* was 1 to<5 cases per 1,000 population. A minority (2.8%) of the population lived in districts with an estimated annual incidence of 5 to<10 *P. falciparum* cases per 1,000 population.

## Discussion

In this study we have developed a modelling approach that combines household and routine HMIS data within a Bayesian hierarchical spatial-temporal model, to compute the annual incidence of *P. vivax* and *P. falciparum* malaria across 398 districts in Afghanistan. The findings demonstrate a strong geographic co-distribution of *P. vivax* and *P. falciparum* malaria morbidity in Afghanistan (Figure 2 and Figure 3). There was no significant change in the mean annual incidence between 2006 and 2009. The incidence of *P. vivax* and *P. falciparum* in 2009 were estimated to be 5.4 and 1.2 per 1000 population respectively. The incidence (for both parasites) was higher in the south-eastern and eastern parts of Afghanistan bordering Pakistan and lowest in northern districts. In addition, the analysis showed that malaria in Afghanistan exhibits a strong seasonal peak between July and November. *P. vivax* tended to peak in August (mean incidence of 7.611 95% Crl 4.849–11.721) compared to *P. falciparum* which peaked in November (mean incidence 2.403 95% Crl 0.929–5.276). However, incidence was low in the winter months between January and May for both parasites. Slightly more than 76% of districts in Afghanistan had predicted incidence of<1 per 1000 population for *P. falciparum* which is a threshold for pre-elimination.

Using the 2006 data as baseline estimates, 17 and 13 provinces had already reduced *P. vivax* and *P. falciparum* incidence respectively by 2009. No reduction in incidence was predicted for Nangahar, Balkh, Sari Pul, Khost and Hirat Nangahar and Khost provinces in south-eastern regions of Afghanistan were amongst those with highest predicted incidence for both parasites. A range of malaria control strategies are implemented at a national level in Afghanistan. LLIN, for example, is targeted in the high to medium risk districts in Badakhshan, Badghes, Baghlan, Balkh, Faryab, Herat, Helmand, Kandahar, Khost, Kunar, Kunduz, Laghman, Nangarhar and Takhar. From the MIS undertaken in 2008, Nangahar had an estimated long lasting insecticidal nets (LLINs) coverage of 19% while no LLINs use was observed in Hirat [37]. Sari Pul region, for example, had some of lowest rates of long lasting insecticidal nets (LLINs) coverage and access to treatment of care. In the districts where indoor residual spraying (IRS) is used as the main vector control approach or to complement LLINs, the targeting of this intervention should be informed by the lag in the peak season of the two main malaria parasites. *P. vivax* peaks in August while *P. falciparum* peaks in November. IRS campaigns should therefore be planned in such away the insecticide are efficacious through the two peak seasons.

Of the 34 provinces of Afghanistan, five (Bamyan, Ghazni, Ghor, Panjsheer and Nuristan) were considered to be malaria free based on altitude thresholds [38]. These provinces, however, accounted for 9.7% of all estimated cases in 2009 indicating a potential problem of importation of malaria cases due to human population movement in Afghanistan or foci transmission in valleys where climatic conditions are favourable. The available data, however, do not provide malaria case definitions and it is impossible to distinguish between imported and local cases. In the malaria free provinces, suspected imported infections should be documented and algorithms, based mainly on travel history, could be used as the basis for case definitions [39]. In addition, health advice and chemoprophylaxis for travellers from the malaria free to endemic provinces should be initiated as an additional package for malaria prevention. An incidence of less than 1 *P. falciparum* case per 1000 individuals is considered to be the threshold for pre-elimination by the WHO [40]. By 2009, 21 provinces in Afghanistan had already achieved such a threshold. However, the biggest challenge is likely to be operational and a comprehensive analysis of overall feasibility of *P. falciparum* elimination [41].

The results of our analysis also have important applications to the design and allocation of resources for malaria case management. Mixed infections especially with *P. vivax* and *P. falciparum* present a challenge for treatment [42]. *P. vivax* infections relapse from dormant liver-stage hypnozoites months after primary infections and are often difficult to diagnose and treat and define as true incident infections [43]. Chloroquine is used as first line treatment of *P. vivax* in Afghanistan as recommended for countries where it remains efficacious and where parasites can be isolated [44], while Artesunate + Sulfadoxine-Pyrimethamine (AS+SP) is used for *P. falciparum* [15]. The incidence maps developed in our study can be used to quantify the need for the number of treatment dosages required for both parasites in Afghanistan. The prevalence of Glucose-6-Phosphate Dehydrogenase (G6PD) deficiency is estimated to be 8% in Afghanistan [45] which complicates the use of the reccomended 14 day regiment of primaquine (PQ) for *P.vivax* [46]. The use of PQ in patients with G6PD deficiency can cause severe haemolysis [47]. To improve the existing information on the prevalence of G6PD deficiency these maps can be used to provide *P. vivax* incidence information that would be useful for the design of G6PD surveys. Where both species are endemic, the use of ACTs has been proposed [48], [49] and other clinical studies have shown a faster parasite clearance rate when ACTs were used [50]. Figure 2 and Figure 3 indicates Kunar, Khost, Laghman, Nangarhar, Takhar and some districts in Kandahar could benefit from such a case management approach.

Although HMIS data used in this analysis represent individuals presenting with fever (symptomatic cases) and with a greater chance of detecting an infection, diagnosis was mainly based on either microscopy or Rapid Diagnostic Test (RDT), both of which have varying sensitivities [51]. In low transmission setting, the rate of false positivity rates, when using RDTs, may be higher and the quality of microscopy in routine HMIS data with varying laboratory conditions may also vary [52]. In addition, it was not possible to identify which diagnosis was used at each facility to adjust the sensitivities. A combination of factors might have limited the effect of environmental covariates especially for *P. falciparum*. This relates to assumptions of linearity during modelling even though rainfall was lagged by four months (SI) and given the short time-series of the data (four years). We used an autoregressive time-varying factor in the model assuming that present state evolves from previous values, but modified by the set of spatial and spatio-temporal covariates[34]. Future studies should relax the linearity assumptions of the fixed effects. Another limitation of the data presented here is that the effects of migration or travel between various regions were not incorporated into the modelling framework. A study in south-eastern Afghanistan showed higher asymptomatic infections in the migrant population [53]. Modelling migration patterns at national level was beyond the scope of this study. It was assumed that individuals would seek treatment at the nearest facility or at least within a district or one of its neighbours. In this study we have modelled malaria morbidity at the facility level and explicitly modelled healthcare attendance at individual facilities as part of our methodology. Security or conflict remains an important factor affecting utilisation of healthcare facilities. This was not modelled in the study due to lack of data, but future studies should consider accounting for this effect on reported health events. Given the additional spatial precision resulting from the facility-level analysis representing febrile individuals within facility catchments, maps of both malaria species are useful for concerted planning.

## Conclusion

This study demonstrates how HMIS and household survey data can be assembled, integrated and interpolated to identify districts with high malaria burden spatially and temporally. Maps were produced at the level of decision-making units, which are potentially useful to the malaria control programme in assessing the changing burden of disease in Afghanistan, targeting malaria interventions at the population most at risk, and planning health resources. The districts identified with high burden should be the focus for targeting vector control. Districts with both *P. vivax* and *P. falciparum* and high rates of mixed infections should be investigated and careful case management strategies adopted. Improved case definition to determine levels of imported risks in malaria free areas is necessary for the elimination ambitions in Afghanistan.

## Supporting Information

File S1 The analysis of public healthcare utilisation for treatment of fever and Bayesian model specification for modelling incidence is provided in the supplementary information. For healthcare coverage and utilisation, this includes the modelling of probability of attendance for fever treatment and delineation of public health facility catchments to estimate population using public health facilities. For incidence modelling, the supplementary information includes details on model specification, parameter estimation and validation.
(DOC)
