# Supplementary material for: Modelling the Incidence of Plasmodium vivax and Plasmodium falciparum Malaria in Afghanistan 2006–2009
Source: PLoS One. 2014 Jul 17;9(7):e102304. doi: 10.1371/journal.pone.0102304 (PMC4102516; doi:10.1371/journal.pone.0102304)
Supplement: File S1 — The analysis of public healthcare utilisation for treatment of fever and Bayesian model specification for modelling incidence is provided in the supplementary information. For healthcare coverage and utilisation, this includes the modelling of probability of attendance for fever treatment and delineation of public health facility catchments to estimate population using public health facilities. For incidence modelling, the supplementary information includes details on model specification, parameter estimation and validation. (DOC) [file pone.0102304.s001.doc]

**Modelling the incidence of *Plasmodium vivax* and *plasmodium falciparum* malaria in Afghanistan 2006-2009**

Victor A Alegana^1, 2^ *, Jim A Wright^2^, Sami M Nahzat^3^ Waqar Butt^4^, Amad W Sediqi^3^, Naeem Habib^4^, Robert W Snow^1, 5^, Peter M Atkinson^2^, Abdisalan M Noor^1, 5*^

**Supplementary Information (SI)**

**Contents**

[1. Introduction 2](#__RefHeading___Toc388034864)

[2. Data 3](#__RefHeading___Toc388034865)

[2.1 Assembly of malaria case data 3](#__RefHeading___Toc388034866)

[2.2 Assembly of environmental or ecological covariates for malaria prediction 6](#__RefHeading___Toc388034867)

[3. Analysis and results 7](#__RefHeading___Toc388034868)

[3.1 Analysis of travel time to health facilities 7](#__RefHeading___Toc388034869)

[3.2 Fever burden and treatment in the public sector 8](#__RefHeading___Toc388034870)

[3.3 Fever and reported rates of utilisation in the Malaria Indicator Survey 9](#__RefHeading___Toc388034871)

[3.4 Distance decay model results for treatment seeking pattern 9](#__RefHeading___Toc388034872)

[4. Extended discussion on healthcare utilisation 14](#__RefHeading___Toc388034873)

[4.1 Modelling healthcare utilisation 14](#__RefHeading___Toc388034874)

[4.2 Uncertainty in modelling utilisation 14](#__RefHeading___Toc388034875)

[5. Bayesian hierarchical models and Gaussian Markov Random Field (GMRF): An overview 15](#__RefHeading___Toc388034876)

[5.1 Bayesian model implementation 17](#__RefHeading___Toc388034877)

[5.2 Model scoring rules 18](#__RefHeading___Toc388034878)

[5.3 Validation Results 19](#__RefHeading___Toc388034879)

# Introduction

This supplementary information provides additional description of datasets for modelling healthcare utilisation and incidence at health facilities. Access to health services continues to be a subject of debate both in high [1,2] and low income countries [3,4]. Poor access to health services has been intertwined with poor health outcomes and poverty. The situation is even more severe in fragile states, such as Afghanistan where majority of population is trapped in vicious circle of poverty, continue to experience low economic development and conflict has lead to destruction to basic infrastructure [5,6].

Previous studies have examined utilisation of health facilities based on demand and supply characteristics. The ability to use a health facility, denoted as potential access in some literature, is usually marked as entry point of studying healthcare access which not only includes the availability of health services but also physical accessibility, affordability, acceptability and system organizational structure [7,8,9]. Of these, physical accessibility is the most studied phenomenon, often using simple straight line distance [10,11] or a combination of spatial and statistical methods [12]. For instance, in Namibia an analysis of 245 facilities indicated that utilisation of the nearest health facilities was 65.3% and earlier studies in Kenya [12,13] and in South Africa [14,15,16], suggested that utilisation of the nearest health facility was as high as 80%. In other studies, where origin and destination data are available, spatial interaction models have been used. Examples include studying commuting behaviour [17], migration [18] and commercial shopping patterns [19].

The main concept underlying the above models is distance-decay phenomenon where utilisation of services decreases with an increasing distance. Distance, however, is just one of the factors that affect utilisation. Other factors include the type and size of health facility, the quality of services offered, socio-economic factors, cultural beliefs, age and sex of individuals and perception of illness [20,21]. For example, Ameli and Newbrander highlight the importance of a female health worker at a health facility and use this as a variable in assessing utilisation [6]. Other studies examining distance have proposed the use of power or exponential functions or log-logistic regression models [22]. A limitation of these studies is the assumption of maximum probability (100%) of use at zero distance [12,14]. Other forms of access studies have used indices such as mean distance to provider, number of services within a pre-defined distance (catchment) or provider-to-population ratios [8,23,24]. A major limitation of these approaches is that they ignore spatial interaction, assume equal utilisation within catchment area, and the calculation of distance is often based on a Euclidean metric [15,25,26,27,28]. Studies that have used a more sophisticated distance metric or travel times have focused on small area geographies or population subgroups with limited national generalisability [29,30,31].

In this study, coverage and utilisation of public health care facilities was assessed using a combination of theoretical travel time to the nearest public health facility and reported patterns of use for treatment of fever. The objectives of the study were to estimate the proportion of Afghan population within a defined public health facility catchment in order to estimate national coverage and the proportion of fever cases within the catchment treated in the public sector in 2011 to understand the level utilisation. The estimated catchment population was subsequently used for analysis of incidence.

# Data

## 2.1 Assembly of malaria case data

Health Management Information Systems (HMIS) form a platform through which information on number of patients passing through a health system is recorded in addition to the type of illness treated [32,33,34]. For malaria this information is vital in estimating case loads through which disease burden can be estimated for planning and allocating health resources.

Error: Reference source not found and Error: Reference source not found (below) show a summary of the assembled case data for *P. falciparum* and *P. vivax* for the 48 month period. Error: Reference source not found includes environmental covariates for enhanced vegetation index (EVI) and precipitation. Precipitation was also lagged by four months to coincide with the peak in cases in July-August period. This data is based on outpatient cases observed at each facility recorded as total malaria cases tested and clinically diagnosed per month. Of the 1,626 facilities, 1,586 had reported both clinical and parasitologically diagnosed cases The slide positivity rate (SPR) was used to adjust the clinical cases, where parasitologically confirmed cases (microscopy or RDT) were reported, to obtain total malaria cases (TMC). It was not possible to distinguish the number of cases diagnosed via microcopy alone or RDT based on the nature of data reported. This is useful to avoid underestimating incidence (if only confirmed cases are used when clinical cases exist) or overestimating incidence (where slide positivity is ignored). Clinical cases were reported at facilities and at health posts linked to sub-health centers.

$$SPR=\frac{Confirmed\begin{matrix} \end{matrix}\begin{matrix} cases \end{matrix}}{Total\begin{matrix} \begin{matrix} \end{matrix}number \end{matrix}\begin{matrix} \begin{matrix} \end{matrix}exa\min ed \end{matrix}}$$

$$TMC=Confirmed\begin{matrix} \end{matrix}\begin{matrix} Case\begin{matrix} \end{matrix}+\begin{matrix} \end{matrix} \end{matrix}\left(Clinical\begin{matrix} \begin{matrix} \end{matrix}cases\begin{matrix} \end{matrix}\times\begin{matrix} \end{matrix}SPR \end{matrix}\right)$$

Overall, Error: Reference source not found below suggests a decline in SPR from 2006 to 2009. For example SPR for hospitals for P*f* was 1.7% in 2006 compared to 0.9% in 2009 and similarly for P*v* (10.1% and 5.0% respectively). A large proportion of reported cases are based on clinical diagnosis from Error: Reference source not found.

Table 2.1: Number of malaria cases by year and type of health facility in Afghanistan.

|  | **Malaria cases (Slide positivity Rate %)** | | | | | | | | | | | |
| --- | --- | --- | --- | --- | --- | --- | --- | --- | --- | --- | --- | --- |
|  | **2006** | | | **2007** | | | **2008** | | | **2009** | | |
| **Type of Facility** | **P*f*** | **P*v*** | **Clinical** | **P*f*** | **P*v*** | **Clinical** | **P*f*** | **P*v*** | **Clinical** | **P*f*** | **P*v*** | **Clinical** |
| **Provincial/Regional Hospitals** | 1,122 (1.7) | 6,800 (10.1) | 10,866 | 1,318 (1.5) | 8,679 (10.0) | 13,636 | 177 (0.4) | 3,658 (8.0) | 5,964 | 950 (0.9) | 5,437 (5.0) | 8,597 |
| **District Hospital** | 562 (0.7) | 10,270 (13.4) | 19,471 | 590 (0.7) | 10,258 (13.0) | 23,038 | 112 (0.3) | 2,273 (6.6) | 15,504 | 508 (0.6) | 8,727 (10.8) | 18,783 |
| **Comprehensive Health Center (CHC)** | 2,481 (1.3) | 38,040 (19.6) | 129,101 | 2,474 (1.1) | 37,134 (16.8) | 141,066 | 571 (0.4) | 17,820 (13.5) | 107,277 | 1,527 (0.8) | 26,295 (13.8) | 108,238 |
| **Sub Health Center (SC)** | 3 (1.2) | 46 (19.01) | 407 | 1 (0.1) | 65 (8.7) | 1,432 | 627 (1.0) | 9,541 (16.7) | 46,675 | 17 (1.8) | 76 (8.0) | 22,292 |
| **Basic Health Center (BHC) (clinics/HPs/MCH)** | 1216 (1.3) | 17,744 (18.3) | 169,272 | 978 (0.8) | 22,823 (19.8) | 200,197 | 2,391 (1.0) | 36,451 (14.4) | 208,949 | 683 (0.5) | 18,898 (14.5) | 165,858 |
| **Total** | **5,384 (1.2)** | **72,900 (16.7)** | **329,117** | **5361 (1.1)** | **78,959 (15.7)** | **379,369** | **3,878 (0.7)** | **69,743 (13.4)** | **384,369** | **3,685 (0.7)** | **59,433 (11.7)** | **323,768** |

Figure 2.1: Assembled cased data and environmental covariates.

Malaria cases at health facilities in Afghanistan by month. The environmental covariates were multiplied by factor (EVI by 1000 and TRMM by 10) for visualization and association with observed cases at the facility. The pattern in each species observed seemed to depict a peak for *P. vivax* at around July-September and October – November for *P. falciparum*.

## 2.2 Assembly of environmental or ecological covariates for malaria prediction

Malaria transmission in Afghanistan is constrained by altitude, temperature [35] and aridity [36,37] which affect parasite sporogony and mosquito vector development [38]. These covariates were assembled from remotely sensed data and extracted for each facility. An average value was extracted for districts without any health facility data and for the facilities (*n=108*) without a geographic reference. All the grid surfaces were resampled to a common resolution of approximately 1 km (cell size 0.008333^o^ x 0.008333^o^).

A temperature suitability index (TSI) [35] rather than the actual temperature values were used. The TSI was modelled from long-term mean monthly temperature data from global climate data (WoldClim, <http://www.worldclim.org/>) [39] and represented optimum mean probabilistic temperature suitability (from 0 (unsuitable) to 1 (most suitable)) for *P. falciparum* and *P. vivax* transmission based on the survival of malaria vectors and on the duration of sporogony (effect on the malaria parasite). Error: Reference source not found (a) shows a classified map of temperature suitability index for Afghanistan.

The monthly enhanced vegetation index (EVI) for the four year period (in Error: Reference source not found) was downloaded from the MODerate-resolution Imaging Spectroradiometer (MODIS) sensor imagery (available at <http://modis.gsfc.nasa.gov/data/>) as measure of vegetation cover [40,41]. A mean index of aridity based on the EVI showed that large areas are under mean annual EVI of <0.1 (Error: Reference source not found (b)). The rate of precipitation was obtained from the Tropical Rainfall Measuring Mission (TRMM 3B43) (<http://trmm.gsfc.nasa.gov/>) [42,43]. TRMM 3B43 (<http://trmm.gsfc.nasa.gov/>) is archived at 0.25^o^ x 0.25^o^ spatial resolution and represent average rate of precipitation in mmhr^-1^ produced after combining satellite data and information from ground stations (rain gauges) [43,44]. The hourly rate was converted to a monthly average based on number of days per calendar month (i.e by multiplying by 24 hours and by 30 days in a month). Error: Reference source not found (c) shows averaged surface for four year period with only few regions recording over 60 mm of rainfall per annum. Only TSI was significant in model selection procedure, however, precipitation and EVI was included in modelling incidence based on expert knowledge from previous studies [45,46]


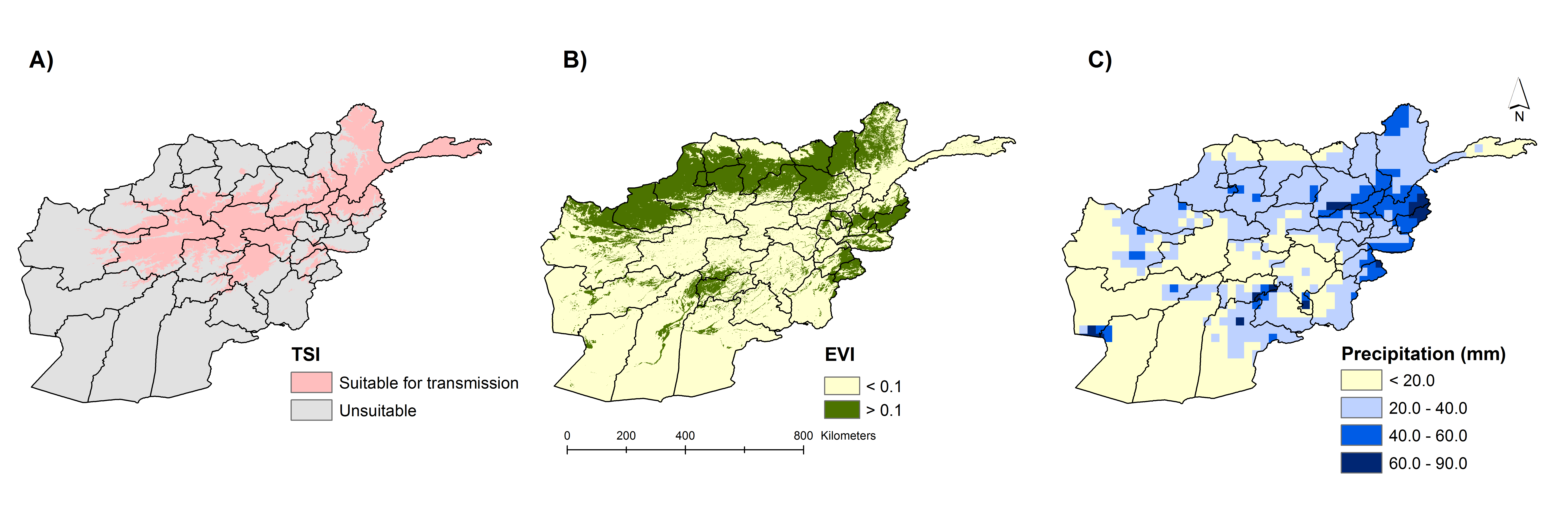


Figure 2.2: Environmental covariates at 1km x 1 km

**A)** Temperature Suitability Index (TSI) for malaria transmission B) the averaged (2006-2009) enhanced vegetation index (EVI) and **C)** the mean precipitation from Tropical Rainfall Measuring Mission (TRMM 3B43).

# Analysis and results

## 3.1 Analysis of travel time to health facilities

This analysis was similar to previous study carried out in Namibia [22] where a combination of ancillary GIS data on land cover, elevation, road and river data layers was used to generate a cost surface of travel times between facilities and population locations in AccessMod (version 3.0) [47]. A friction surface was generated based on cumulative travel speed between patient origins (households) and destinations (public health facilities) at 1 km by 1 km grid resolution. Travel speeds used for the analysis provided in (Error: Reference source not found below) were assigned to each land cover pixel based on recommendations from previous similar studies [22,29,47]. The derived friction surface was used to extract travel times between cluster locations and health facilities using ArcGIS (ESRI, Redlands, CA). Thus, all the individuals in a cluster were assigned an average community travel time to the nearest public health facility (SI 2). Since all age-cohort data were available, a preliminary analysis that included age (regression coefficient -0.0155, 95%CI: -0.0314 – 0.0097, p=0.07), gender (regression coefficient -0.3327, 95%CI: -0.9516 – 0.2704, p=0.2830) and derived travel time (regression coefficient -0.0860, 95%CI: -0.3986 – -0.0563, p<0.001) suggested that only the latter had an effect on the use of public health facilities. Thus, age and sex were subsequently dropped when modelling the distance decay curves. A three parameter logistic regression model of the form $Y=\kappa/(1+e^{(\alpha-x)/\beta})$ [48] was then fitted to predict probability of health facility attendance based on the extracted theoretical travel times, assuming individuals visited the nearest health facility (SI 2). Four models representing the universe of all public health facilities, hospitals (District, regional and national referral), health centres (sub-health centres and CHC) and basic facilities (MCHs, clinics and HPs) were fitted separately to the survey data in the R statistical software [49]. The model coefficients ($\alpha$ an asymptote factor at an inflection point; $\beta$, a distance decay parameter; and $\kappa$, a limiting function on the *y*-axis that measured the probability of attendance when distance is zero) were recorded along with the goodness-of-fit statistic, *t*, and the *p*-values.

Table 3.2: Travel speeds

Description of various data sources used as inputs in calculating travel time to the active public health facilities in Afghanistan. The assumed travel speeds for each input feature are also shown.

| **Map Layer** | **Description** | **Classification** | **Speed (km/h)** | **Mode^1^** |
| --- | --- | --- | --- | --- |
| Land use/ land cover | Spatial representation of all different land use and land cover types. Two land cover grids were processed (1) a basic land cover grid (2) a combined grid that incorporates roads and rivers with the same resolution as the DEM | Irrigated, rain fed, mosaic or vegetated croplands | 3.0 | Walking |
|  |  | Open or closed broadleaved, needle leaved, deciduous or evergreen tree cover | 3.0 | Walking |
|  |  | open or closed mixed broadleaved forest/tree cover | 1.5 | Walking |
|  |  | Mosaic, closed to open grassland/shrubland | 1.5 | Walking |
|  |  | Sparse Vegetation | 1.5 | Walking |
|  |  | Open or closed broadleaved regularly flooded | 0.5 | Walking |
|  |  | Artificial/urban areas | 30.0 | None |
|  |  | Bare areas/desert | 1.0 | Walking |
|  |  | Ice/ permanent snow | 0 | None |
| Roads | Classified into five categories; class A (highways), class B (secondary roads), tertiary Class C and Class D roads as well as street level urban roads. Each road class was assigned a different speed limit. | Class A roads | 60.0 | Motorised |
|  |  | Class B roads | 30.0 | Motorised |
|  |  | Class C roads | 10.0 | Cycling |
|  |  | Class D roads | 4.0 | Walking |
|  |  | Street level roads in urban areas | 20.0 | None |
| Rivers | GIS layer representing barrier to movement. Only major rivers were used to reduce the complexity of running the algorithms | NA^3^ | 0 | NA^2^ |
| Digital elevation model | Altitude values that are used in anisotropic calculation; Original DEM 30 m ASTER grid; resampled to 1 km pixel size | Degree of Slope (< 0.5^o^) | 4.88 | Walking |
|  |  | Degree of Slope (5.0^o^) | 3.71 | Walking |
|  |  | Degree of Slope (10.0^o^) | 2.71 | Walking |
|  |  | Degree of Slope (20.0^o^) | 1.41 | Walking |
|  |  | Degree of Slope (30.0^o^) | 0.66 | Walking |

1. Assumed mode of travel to health facility, as either walking on foot, cycling, using motorise transport as on roads or a combination of the different modes. Anisotropic movement for walking based on Tobler’s equation, (*V*=6*exp(-3.5abs[Tan(slope in degrees/57.296) + 0.05]) [50] where *V* is the speed with slope derived from DEM or for cycling [51], was applied for traversing across a pixel. For example, on a flat terrain, the walking speed is 5.0 km hr^-1^.
2. NA is an abbreviation for ‘ Not Applicable’

## 3.2 Fever burden and treatment in the public sector

The rate of fever reported from the MIS at province level was applied to all the constituent catchment populations within each province to generate a fever burden map. The number of fever cases within derived catchment was extracted from the fever burden map while the number of fever cases likely to attend a public health facility was calculated by multiplying the estimated number of cases by the probability of attendance.

## 3.3 Fever and reported rates of utilisation in the Malaria Indicator Survey

15,442 individuals in 183 clusters were interviewed in the 2011 Afghan MIS. Fever prevalence was estimated as 2.1% (95%CI: 1.8 – 2.3, *n* = 327) in all age populations in 2011, of which 84.1% (95%CI: 80.1 – 88.7) sought some form of treatment from either the formal or informal sector. Of those who sought treatment, a majority (44.7%, 95%CI: 38.8 – 50.6) used the public sector compared to 42.2% (95%CI: 36.3 – 48.1) who used the private sector comprising private clinics, private hospitals or drug stores.

## 3.4 Distance decay model results for treatment seeking pattern

Error: Reference source not found (below) shows the modelled probability of attendance for fever by different types of public health facility in relation to travel time for Afghanistan. Error: Reference source not found lists the various parameters for each fitted model along with their respective *p*-values and related goodness-of-fit statistics. In three of the four fitted models, probability of attendance for fever treatment decayed rapidly after 125 minutes (approximately 2 hours) except for the hospitals where decay was rapid after 175 minutes (approximately 3 hours). The coefficients of the various decay curves were all significant with *p*<0.001 with the sum of squared residuals suggesting a good model fit with the observed fever treatment patterns. From the distance decay curves, the treatment-seeking pattern for the basic health facilities was similar to that of the health centres. The universal *“all”* model was an average of the three models. The modelled pattern suggested a longer travel time (slower decay) in the utilisation of hospitals. In addition, the maximum probability of use was slightly larger for HCs (0.877) and for the basic health facilities (0.881) compared to the hospitals (0.870). The universal model was subsequently used to delineate catchments and derive the catchment population. This model produced smaller values of the sum of squared residuals and standard error compared to the other three models.

 A probability surface of attendance for fever treatment at all health facilities was derived at 1 km by 1 km resolution (see supplementary information) and used subsequently to estimate the catchment population for the analysis of incidence. In summary, of the estimated population (32.3 million) in 2011, 27.8 million (85.8%) were estimated to be within 2 hours’ of travel of a public health facility and, therefore, within its catchment (supplementary information). Further, 17.9 million (64.4%) of those within a public health facility catchment were within 30 minutes and 13.1 million (47.4%) were within distances where the probability of attendance was ≥60%. 9,071,360 (32.6%) had much lower probabilities of attendance (≤0.20).

Error: Reference source not found shows the modelled probability of attendance for fever by different types of public health facility in relation to travel time for Afghanistan and subsequently catchment map for public health facilities (Error: Reference source not found). Of the estimated population (32.3 million) in 2011, 27,784,873 (85.8%) were estimated to be within 2 hours’ of travel of a public health facility and therefore within its catchment (Error: Reference source not found below). Further, 17,890,000 (64.4%) of those within a public health facility catchment were within 30 minutes and 13,158,318 (47.4%) were within distances where the probability of attendance was ≥60%. 9,071,360 (32.6%) had much lower probabilities of attendance (≤0.20). Based on the MIS-derived fever prevalence, the estimated national fever burden was 327,517 cases assuming a single episode of fever in October 2011 and from the distance decay curves for facility attendance, 300,427 (91.7%) of these cases were within 2 hours’ travel time to the nearest public health facility (Table 3.3). Finally, 145,085 (44.3%) of these fever cases were likely to have been treated in the public sector based on the distance decay model.

Table 3.3: Parameters of logistic regression model.

Parameters of the logistic model for active health facilities (*n* = 1,581) in Afghanistan by type. The parameter $\alpha$ represent model asymptote, $\beta$as distance decay parameter and $k$ as scaling factor.

|  |  | Model Parameter | | |  |  |  |
| --- | --- | --- | --- | --- | --- | --- | --- |
|  | **Number of facilities** |  |  |  | **p-value (all parameters)** | **Residual standard error** | **Sum of Squared residuals** |
| All | 1,581 | 3.1906 | -0.2908 | 0.8681 | <0.000 | 0.0021 | 0.0015 |
| Hospitals | 129 | 3.8439 | -0.3139 | 0.8702 | <0.000 | 0.0218 | 0.0176 |
| Health Centres | 698 | 2.8896 | -0.2863 | 0.8768 | <0.000 | 0.0182 | 0.0121 |
| Basic | 754 | 2.8066 | -0.2843 | 0.8805 | <0.000 | 0.0175 | 0.0113 |


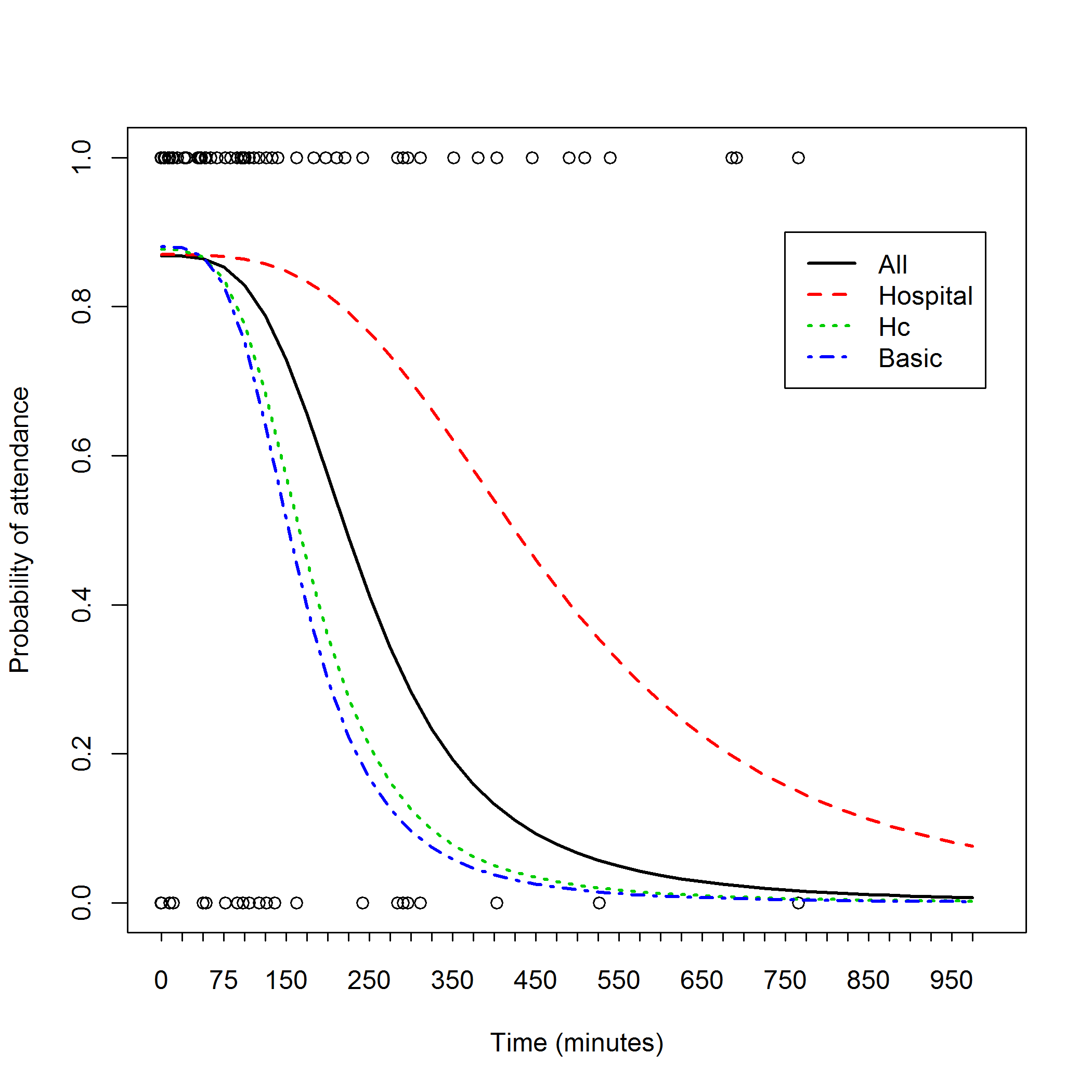


Figure 3.3: Distance decay curves.

Distance decay curves for the MIS survey (2011) showing probability of public health facility use for fever treatment in Afghanistan (*y*-axis) against increasing travel times (*x*-axis). The model was run using log-transformed travel time (*x*-axis) then back-transformed for presentation purposes. The attendance pattern (1 = attendance and 0 = non-attendance) is also superimposed on the decay curve.


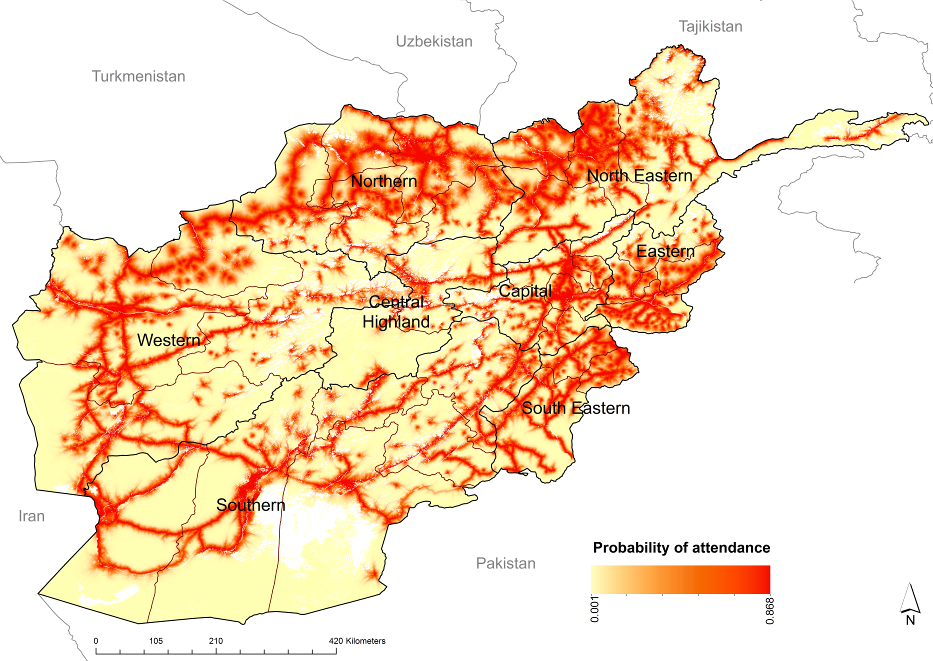


Figure 3.4: Probability of attendance.

Map of probability of attendance for fever treatment at the nearest public health facility for all age cohorts in Afghanistan, based on the 2011 MIS survey. The lowest probability was 0.001 and the highest probability was 0.868.


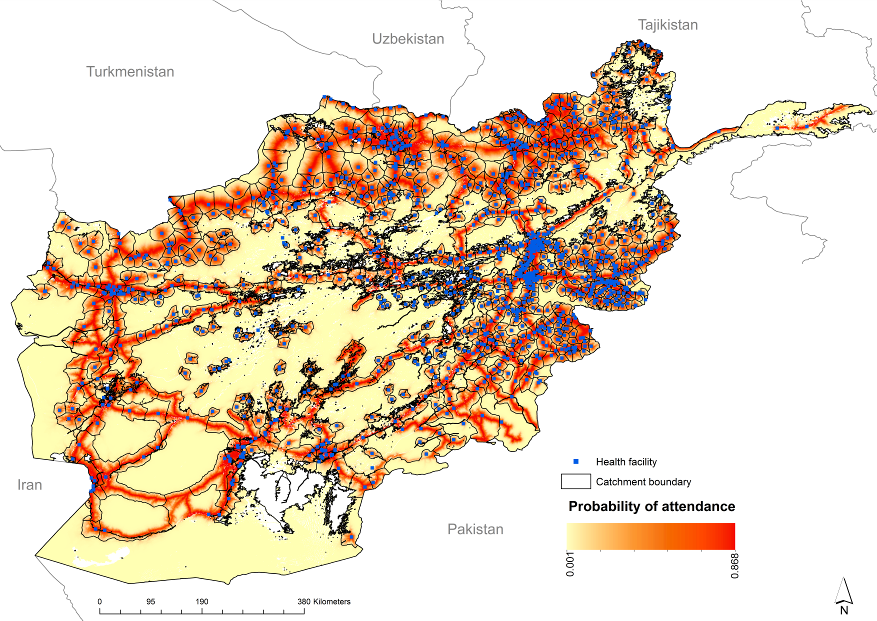


Figure 3.5: Health facility catchments

Map of public health facility catchment areas for Afghanistan, derived from modelled travel time (superimposed on the probability of attendance at nearest public health facility when sick with fever).

Table 3.4: Estimated population data for 2011 by province and modelled treatment seeking for fever at the nearest public health facility.

|  | **Hospitals (Provincial/Regional/District)** | **Health Centers (Comprehensive and sub health Centers)** | **Basic Health facility (HPs/Clinics/MCH)** | **Other Facilities** | **Total health facilities** | **Estimated Population in 2008 (All ages)** | **Population (percentage) in PHF^1^ catchments** | **Estimated fever burden for 2011 from MIS prevalence** | **Number of fever cases likely to attend a PHF^1^ (%)** |
| --- | --- | --- | --- | --- | --- | --- | --- | --- | --- |
| **Probability of attendance** |  |  |  |  |  |  |  |  |  |
| ≤0.20 | 15 | 93 | 117 | 2 | 227 | 13,649,346 | 9,071,360 | 124,967 | 5,931 (4.7) |
| >0.20 -< 0.50 | 5 | 26 | 45 | 4 | 80 | 3,267,810 | 3,267,810 | 39,725 | 13,124 (33) |
| >0.50 -< 0.60 | 5 | 9 | 15 | 1 | 30 | 2,287,390 | 2,287,390 | 28,231 | 16,628 (58.9) |
| ≥ 0.60 | 104 | 570 | 577 | 41 | 1292 | 13,166,100 | 13,158,313 | 134,595 | 109,402 (81.3) |
| **Travel time** |  |  |  |  |  |  |  |  |  |
| ≤ 30.00 minutes | 121 | 661 | 708 | 48 | 1538 | 17,890,000 | 17,890,000 | 192,425 | 136,890 (71.1) |
| >30.00 min - <1.00 hours | 7 | 27 | 28 | 0 | 62 | 23,306,950 | 23,306,950 | 66,141 | 7,495 (11.3) |
| >1.00 hours -< 2.00 hours | 1 | 9 | 16 | 0 | 26 | 27,792,610 | 27,792,610 | 41,906 | 672 (1.6) |
| ≥ 2.00 hours | 0 | 1 | 2 | 0 | 3 | 4,578,036 | - | 27,045 | 28 (0.1) |
| **Province** |  |  |  |  |  |  |  |  |  |
| Badakhshan | 3 | 35 | 34 | 1 | 73 | 1,351,920 | 974,008 | 11,324 | 1,749 (15.4) |
| Badghis | 2 | 10 | 23 | 0 | 35 | 584,251 | 488,069 | 10,897 | 2,222 (20.3) |
| Baghlan | 3 | 30 | 21 | 0 | 54 | 1,101,920 | 925,927 | 5,399 | 1,754 (32.4) |
| Balkh | 7 | 41 | 46 | 3 | 97 | 1,493,720 | 1,456,970 | 20,686 | 12,427 (60.0) |
| Bamyan | 4 | 27 | 18 | 1 | 50 | 534,916 | 288,573 | 34 | 3 (9.8) |
| Day Kundi | - | - | - | - | - | 578,854 | 88,678 | 20,689 | 595 (2.8) |
| Farah | 2 | 21 | 6 | 1 | 30 | 615,616 | 402,153 | 0 | 0 |
| Faryab | 3 | 26 | 22 | 0 | 51 | 1,153,790 | 1,048,770 | 25 | 4 (17.4) |
| Ghazni | 4 | 31 | 35 | 1 | 71 | 1,527,840 | 1,244,250 | 19 | 1 (6.2) |
| Ghor | 3 | 25 | 21 | 0 | 49 | 814,963 | 375,385 | 27 | 0 |
| Hilmand | 4 | 25 | 27 | 1 | 57 | 1,100,790 | 805,764 | 6 | 0 |
| Hirat | 5 | 42 | 40 | 1 | 88 | 2,221,470 | 1,973,640 | 12 | 1 (4.2) |
| Jawzjan | 4 | 13 | 16 | 0 | 33 | 665,411 | 644,905 | 8,974 | 4,036 (44.9) |
| Kabul | 41 | 39 | 61 | 25 | 166 | 4,872,250 | 4,840,000 | 20,601 | 16,365 (79.4) |
| Kandahar | 2 | 18 | 18 | 2 | 40 | 1,460,940 | 1,168,280 | 0 | 0 |
| Kapisa | 2 | 17 | 16 | 2 | 37 | 488,739 | 466,414 | 33 | 8 (24.6) |
| Khost | 1 | 19 | 9 | 1 | 30 | 697,456 | 696,841 | 3,535 | 1,750 (49.4) |
| Kunar | 1 | 18 | 13 | 0 | 32 | 547,826 | 532,095 | 42,121 | 14,645 (34.7) |
| Kunduz | 2 | 17 | 32 | 2 | 53 | 1,203,720 | 1,177,470 | 13,974 | 7,306 (52.2) |
| Laghman | 1 | 21 | 16 | 0 | 38 | 535,115 | 504,745 | 9,610 | 3,776 (39.2) |
| Logar | 3 | 11 | 20 | 0 | 34 | 479,402 | 432,285 | 21 | 2 (8.8) |
| Nangarhar | 6 | 33 | 63 | 1 | 103 | 1,828,820 | 1,751,860 | 119,758 | 63,249 (52.8) |
| Nimroz | 1 | 9 | 5 | 1 | 16 | 198,911 | 139,575 | 0 | 0 |
| Nuristan | 0 | 12 | 12 | 0 | 24 | 187,256 | 136,591 | 77 | 8 (10.0) |
| Paktika | 3 | 10 | 18 | 0 | 31 | 538,622 | 475,151 | 6 | 1 (15.2) |
| Paktya | 3 | 17 | 17 | 0 | 37 | 683,023 | 675,139 | 4,851 | 2,116 (43.6) |
| Panjshir | 1 | 6 | 4 | 0 | 11 | 150,659 | 88,775 | 4 | 0 |
| Parwan | 2 | 31 | 32 | 5 | 70 | 884,168 | 774,160 | 6,792 | 3,545 (52.1) |
| Samangan | 2 | 15 | 13 | 0 | 30 | 467,796 | 391,992 | 16 | 2 (15.3) |
| Sari Pul | 3 | 14 | 16 | 0 | 33 | 723,273 | 632,692 | 8,584 | 2,288 (26.6) |
| Takhar | 4 | 21 | 38 | 0 | 63 | 1,194,430 | 1,149,240 | 7,276 | 4,031 (55.4) |
| Uruzgan | 1 | 7 | 6 | 0 | 14 | 428,158 | 296,071 | 11 | 1 (6.0) |
| Wardak | 4 | 28 | 27 | 0 | 59 | 683,159 | 518,201 | 12,157 | 3,200 (26.3) |
| Zabul | 2 | 9 | 9 | 0 | 20 | 371,462 | 220,204 | 0 | 0 |
| **Total** | **129** | **698** | **754** | **48** | **1629** | **32,370,646** | **27,784,873^2^** | **327,517** | **145085(44.3)** |

1. PHF is abbreviation for Public Health Facility
2. The total number of people in the catchment was lower than the overall estimated population because some population were outside the catchment boundary, thus not covering 100% population and not entire population is likely to use a PHF.

# Extended discussion on healthcare utilisation

## 4.1 Modelling healthcare utilisation

The analysis of utilisation suggested an improvement in the coverage of BPHS by 2011 with 85.8% of the population estimated to be within the health facility catchments. The results reported by the national Afghanistan Health Survey (AHS) conducted in 2006 indicated majority (75%) walk to a health facility, 92% use only one mode of transport and estimated over 60% of the rural population to be within 2 hours of a health facility [52]. The MIS of 2011 was conducted in the September-October period and the reported two-week-period prevalence of fever was low at 2.1% (95%CI: 1.8 – 2.3, *n* = 327) with reported rates of utilisation of the public sector for fever treatment at 44.7% (95%CI: 38.8 – 50.6), similar to overall use of the private sector at 42.2% (95%CI: 36.3 – 48.1). Our utilisation rates from the distance decay model predicted that 44.3% of the fever burden in 2011 was likely to have been treated in the public sector. Thus, the predicted rate of utilisation in the public sector was similar to the observed utilisation rate based on the MIS survey. However, the rate of utilisation declined rapidly after about 120 minutes regardless of the facility type (Error: Reference source not found). The rate of decline was also rapid for basic health facilities compared to that from previous studies [12,22] which could reflect a reluctance to travel longer distances for security reasons, cost or due to gender and cultural norms [53]. A study conducted in Kabul by Mashal *et al*., (2008) identified that factors such as mothers’ lack of autonomy in the household and low level of education inhibited treatment-seeking behavior, resulting in poor health outcomes such as under-nutrition or acute respiratory infections [54]. The modelled pattern also suggested that the population is likely to travel greater distances for hospital-based services and slightly higher probabilities of use at zero distance were observed for basic health facilities $\kappa$(0.881, *p*<0.001) and for the health centres $\kappa$(0.877, *p*<0.001) (Error: Reference source not found and Error: Reference source not found above). This phenomenon could be attributed to proximity of the basic health facilities to population and the tendency to use basic facilities for uncomplicated illness especially for maternal and child services [55].

## 4.2 Uncertainty in modelling utilisation

Variation in travel modes could also affect the pattern of use, as would perception on quality of healthcare services [56,57]. Empirical data to test these assumptions are not readily available at the national level and analysis was restricted to interaction with the closest public health facility. Additionally, utilisation rates were estimated from cross-sectional surveys and, therefore, may not capture temporal changes in care-seeking behaviour over the four year period.

Some sources of errors remain while modelling utilisation besides those discussed. These include the exclusion of factors explaining health facility utilisation such as household income, healthcare costs, wealth and cultural preferences [12,15,58,59,60,61,62]. Access, as a multidimensional concept, is affected by these factors. Although the household data on reported rates of fever and treatment seeking behaviour were representative for all ages, the inclusion of the above factors could alter the probability of using the nearest health facility even though the reported use (from the MIS) of the public sector was similar to that predicted by the model. Since this study has focused mainly on patients’ interactions with the public health sector, the inclusion of private sector facilities may well alter the patterns of use observed here. Additionally, the different modes of transport such as walking or use of motorized transport could differ from those assumed in the model. Data on actual mode of transport used while travelling to a health facility are rarely available.

Further, the calculated fever burden was based on a survey-derived regional period prevalence rate, when point prevalence may vary at community or facility catchment level [63,64]. This fine resolution point prevalence may be significantly different from the regional mean. Lastly, the study did not account for the effects of conflict while modelling utilisation. It was assumed that conflict effects were inherent in respondent answers at the survey stage. Secondly, such effects required a time series of data on incidences of violence to identify stable hotspot areas that was beyond the scope of this study. Future studies could investigate the probability of conflict as a variable in determining probability of health facility use in fragile provinces.

# Bayesian hierarchical models and Gaussian Markov Random Field (GMRF): An overview

GMRFs are widely used in Bayesian hierarchical models applications due to their relative ease of implementation and Markovian properties [65]. They are useful at representing dependence of unobserved process (the latent effect) at the second stage of a hierarchical Bayesian model. The first level of a hierarchical model relates to observations (the data) and its distribution properties. Thus, for example, he observed malaria cases $y_{i}(i=1,......n)$ are believed to exhibit a Poisson distribution. A random vector $x=(x_{1},........x_{n})^{T}$can be defined as a GMRF with mean $\mu$ and positive definite precision matrix$Q$ with density as:

$$\pi(x)=2\pi^{-n/2}|Q|^{1/2}\exp[-\frac{1}{2}(x-\mu)^{T}Q(x-\mu)]$$

where $\pi$ is the density and $Q$ is the precision matrix with covariance matrix $\Sigma=Q^{-1}$. Two random variables (RV) $x_{i}$ and $x_{j}$ $(i\neq j)$are conditionally independent with conditional density $\pi(.|.)$ if$\pi(x_{i}\begin{matrix} x_{j} \end{matrix}|x_{-ij})=\pi(\begin{matrix} x_{i} \end{matrix}|x_{-ij})\cdot\pi(\begin{matrix} x_{j} \end{matrix}|x_{-ij})$. For a GMRF, $Q$ is usually sparse allowing for fast computation based on Cholesky decomposition in Integrated Nested Laplace Approximation (INLA) see [65]. The joint posterior distribution$\pi(y|x,\theta)$, where $y$ are the observations,$x$ latent Gaussian variable with $\theta$ hyperparameters can be written as:

$$\pi(x,\begin{matrix} y| \end{matrix}\theta)\begin{matrix} \end{matrix}\alpha\begin{matrix} \end{matrix}\pi(\theta)\begin{matrix} \pi(x|\theta) \end{matrix}\begin{matrix} \prod\pi(y_{i}|x_{i},\begin{matrix} \theta\end{matrix}) \end{matrix}$$

$$\alpha\begin{matrix} \end{matrix}\pi(\theta)\begin{matrix} \end{matrix}\begin{matrix} |Q|^{1/2}\exp[-\frac{1}{2}x^{T}\begin{matrix} \end{matrix}Q(\theta)\begin{matrix} \end{matrix}x\begin{matrix} +\begin{matrix} \sum\log\left\{\pi(y_{i}\right. \end{matrix} \end{matrix} \end{matrix}|x_{i},\left.\theta\right\}$$

For Bayesian inference the desired marginal distribution is

$$\pi(x_{i}|y_{i})\begin{matrix} \end{matrix}=\begin{matrix} \end{matrix}\int\pi(x_{i}|\theta,\begin{matrix} y)\begin{matrix} \pi(\theta|y)\begin{matrix} d\theta\end{matrix} \end{matrix} \end{matrix}$$

$$\pi(\theta_{j}|y)\begin{matrix} \end{matrix}=\begin{matrix} \end{matrix}\int\pi(\theta|y)\begin{matrix} \end{matrix}d\theta_{-j}$$

which is approximated in INLA when the unknown parameters are not very large ($\theta$ is small typically less than 10) [66,67,68]. The INLA method, arrives at a closed solution using curvature of the mode evaluated at suitable sampling points [67]. The difference between INLA and widely used MCMC [69] relates to computational efficiency that the former enjoys. INLA however applies for a class of Latent Gaussian Models (LGM) such as spatial and spatio-temporal models.

The integral is then evaluated via a finite sum

With $\Delta_{k}$as weights at appropriate values $\theta_{k}$. The approximation of $\pi(\theta|y)$ is obtained by integrating $\theta_{-j}$from $\tilde{\pi}(\theta_{j}|y)$[66,70]. The initial stage involve finding the mode at supporting points followed by a laplace approximation of $\tilde{\pi}(x_{i}|\theta,y)$ to the conditional marginal distribution to $\pi(x_{i}|\theta,y)$

where $\tilde{\pi}G(x|\theta,\begin{matrix} y) \end{matrix}$is the Gaussian approximation to conditional of $x$ evaluated at the mode$x^{*}(\theta)$obtained by optimization algorithm using quasi-Newton approach [68] and

## 5.1 Bayesian model implementation

A zero-inflated Poisson model was implemented based on observed counts of cases for *P. falciparum* and *P. vivax* as

$$Y_{ij}\sim\left\{\begin{matrix} 0 \\ Poisson(\mu_{ij}) \end{matrix}\right.\begin{matrix} \\ \end{matrix}\begin{matrix} \\ \end{matrix}\begin{matrix} \\ \end{matrix}\begin{matrix} with\begin{matrix} probability\begin{matrix} P_{ij} \end{matrix} \end{matrix} \\ with\begin{matrix} probability\begin{matrix} (1-P_{ij}) \end{matrix} \end{matrix} \end{matrix}$$

where $Poisson(\mu_{ij})\begin{matrix} =\begin{matrix} \exp(-\mu_{ij}) \end{matrix} \end{matrix}\mu_{ij}^{y_{ij}}/y_{ij}!$ with the $\log it(p_{ij})=\log[p_{ij}/(1-p_{ij})]$.

The general Bayesian regression model was represented as

$$\log\begin{matrix} \mu_{i} \end{matrix}=\begin{matrix} \end{matrix}\log(E_{i})\begin{matrix} \end{matrix}+\begin{matrix} \end{matrix}\alpha\begin{matrix} \end{matrix}+\begin{matrix} \end{matrix}X_{ij}^{T}\beta_{ij}\begin{matrix} \end{matrix}+\begin{matrix} \end{matrix}f(s_{u})\begin{matrix} \end{matrix}+\begin{matrix} \end{matrix}\phi_{i}\begin{matrix} \end{matrix}+\begin{matrix} \end{matrix}f(t)$$

where *E_i_* was the expected number of cases adjusted for utilisation at each facility *i*, $\alpha$ as the intercept, with the$f_{unstr}(\cdot)$ terms representing the spatially unstructured effects at facility, district and province levels while $f_{t}(.)$ represented the seasonal or temporal effects. Thus, the likelihood of the data assuming similar covariates for the zero-state and the Poisson state as

$$\begin{matrix} L_{i}(\theta,\begin{matrix} Z|y,\begin{matrix} \alpha)\begin{matrix} =\begin{matrix} \prod_{j=1}^{Ei} [p_{ij} \end{matrix} \end{matrix} \end{matrix} \end{matrix}\Pr(Y_{ij}\begin{matrix} =\begin{matrix} 0|Z_{ij}\begin{matrix} =\begin{matrix} 1,\begin{matrix} \theta)]^{Z_{ij}} \end{matrix} \end{matrix} \end{matrix} \end{matrix} \end{matrix} \\ \begin{matrix} \begin{matrix} & & & & \times\end{matrix} & [(1\begin{matrix} -\begin{matrix} p_{ij} \end{matrix} \end{matrix})\begin{matrix} \Pr(Y_{ij}\begin{matrix} =\begin{matrix} y_{ij} \end{matrix} \end{matrix}|Z_{ij}\begin{matrix} =\begin{matrix} 0 \end{matrix} \end{matrix},\begin{matrix} \end{matrix}\alpha,\begin{matrix} \end{matrix}\theta)]^{1-Z_{ij}} \end{matrix} & & \end{matrix} \end{matrix}$$

Inverse Gamma priors $IG(a,b)\begin{matrix} a=1.0,\begin{matrix} b=0.005 \end{matrix} \end{matrix}$ were assigned to precision hyperparameters $\tau^{2}$ for the unstructured effects components$\theta_{unstr}\sim N(0,\tau^{2})$ at facility and district and province level. For the temporal trend, a first-order auto-regressive process process,$\rho Y(s_{i},t_{i-1})$ with the first term coming from a stationary distribution $N(0,\sigma_{w}^{2}\sum)$that depends on past values $x_{i}\begin{matrix} =\begin{matrix} \rho\begin{matrix} \end{matrix}x_{i-1}\begin{matrix} +\begin{matrix} \xi_{i} \end{matrix} \end{matrix} \end{matrix} \end{matrix}\begin{matrix} & \xi_{i}\sim N(0,\tau^{2} \end{matrix})$for $0>\rho<1$ was assigned [71]. The conditional-autoregressive prior was used as a spatial effect at the district level. The conditional prior for neighbouring districts $(\phi_{j},\begin{matrix} \end{matrix}j\neq i)$ was specified similarly to Bernardinelli *et al.,* (1997) as $(\phi_{i}\sim N(\mu_{\varphi i},\sigma_{\varphi i}^{2})$ where $\mu_{\varphi i}=\Sigma_{j\neq i}W_{ij}\phi_{j}/\Sigma_{j\neq i}W_{ij})$ ; $\sigma_{\phi i}^{2}=1/\gamma_{\phi}\Sigma_{j\neq i}W_{ij})$ [72]. The *W_ij_* represented an adjacency matrix of weights assigned as *W_ij_* = 1 for two neighbouring regions or *W_ij_* = 0 otherwise. Non informative priors $\pi(\theta)\begin{matrix} \end{matrix}\alpha\begin{matrix} \end{matrix}1$ were assigned on the fixed covariate effects. The model was run in R-INLA [67,73] (available at <http://www.r-inla.org/> ). The posterior taking into account of the priors$\pi\{\theta\left(\beta_{i},\begin{matrix} \tau_{i},\begin{matrix} \phi,\begin{matrix} \rho\end{matrix} \end{matrix} \end{matrix}\right)\}$,

$$\begin{matrix} f(\theta,\begin{matrix} \alpha,\begin{matrix} Z \end{matrix}|y\begin{matrix} )\begin{matrix} =\prod_{i=1}^{m} \begin{matrix} \left\{\prod_{j=1}^{n_{i}} [P_{ij}^{Z_{ij}}[(1-P_{ij})\right. \end{matrix} \end{matrix} \end{matrix} \end{matrix}\begin{matrix} \Pr\begin{matrix} (Y_{ij} \end{matrix} \end{matrix}\begin{matrix} =y_{ij} \end{matrix}|Z_{ij}=0),\begin{matrix} \theta\end{matrix}]^{1-Z_{ij}} \\ \begin{matrix} & & & \end{matrix}\begin{matrix} \end{matrix}\begin{matrix} \times\begin{matrix} \left|\Psi_{\beta}\right| \end{matrix} \end{matrix}^{-\frac{1}{2}}\exp\left((-\frac{1}{2}(\beta-\beta_{0})^{'}\Psi_{\beta}^{-1}(\beta-\beta_{0})\right) \\ \begin{matrix} & & & \end{matrix}\begin{matrix} \end{matrix}\times\begin{matrix} \sigma^{-a} \end{matrix}\exp\left(\frac{-b}{2\sigma^{2}}\right) \\ \begin{matrix} & & & \end{matrix}\begin{matrix} \end{matrix}\times\begin{matrix} \Pi\left(\theta\right) \end{matrix} \end{matrix}$$

## 5.2 Model scoring rules

There are different methods to evaluate of model uncertainty. One approach is to score based on the probabilistic values from predictive distribution of the model compared to actual observations [74]. The score is said to be proper if there is consistency between the predictions and the observations (model is correctly calibrated). Thus the two competing models do not need to be related and can be independent as long as there is correct calibration and an assessment of sharpness [75]. Gneiting and Raftery (2007) reviews some of the proper scoring rules including the use of standard error score (SES), the Dawid-Sebastiani score (DSS), the logarithmic score (logS), and the ranked probability score (RPS). The two computed scores in this study were the DSS and SES given by:

$$SES(P,\begin{matrix} y \end{matrix})\begin{matrix} =\begin{matrix} (y-\mu P) \end{matrix} \end{matrix}^{2}$$

$$DSS(P,\begin{matrix} y \end{matrix})\begin{matrix} =\begin{matrix} \frac{1}{2}\cdot\left(\log\left(\sigma^{2}\right)\begin{matrix} +\begin{matrix} \left(\left(y\begin{matrix} -\begin{matrix} \mu_{p} \end{matrix} \end{matrix}\right)/\sigma_{p}\right)^{2} \end{matrix} \end{matrix}\right) \end{matrix} \end{matrix}$$

where $P$ is the predictive distribution with mean $\mu$and standard deviation$\sigma_{p}$ [74]. SES is faily similar to the mean square error (MSE) but for a predictive distribution. The DSS is proper for latent Gaussian models[74].

The leave one out cross validation score using the conditional predictive ordinate (CPO) was also evaluated. For CPO, a prediction is validated based on the predictive distribution and the remaining data only [76,77]. Thus it is the probability of observing a value given all other data. No data values for the fitted model failed CPO test which is likely to happen if the approximation of the latent Gaussian Field (GF) is not sufficiently accurate [76]. To predict a value $y_{p}$given other values, the predictive density $P(y_{p}|y_{-p})$ is given by:

$$P(y_{p}|y_{-p})\begin{matrix} =\begin{matrix} \int\pi(y \end{matrix} \end{matrix}|\theta,\begin{matrix} y_{-p} \end{matrix})\begin{matrix} \pi(\theta|y_{-p} \end{matrix})\begin{matrix} d\theta_{p} \end{matrix}$$

and obtained via a finite sum with weights $\Delta_{j}$:

$$P(y_{p}|y_{-p})\begin{matrix} \approx\begin{matrix} \sum_{1}^{j} \pi(y_{p} \end{matrix} \end{matrix}|\theta_{j},\begin{matrix} y_{-p} \end{matrix})\begin{matrix} \pi(\theta_{j} \end{matrix}|y_{-p})\begin{matrix} \Delta_{j} \end{matrix}$$

The correlation coefficient and the mean error (ME) was used to provide an association of the predicted incidence with the crude observed incidence at district level. Scatter plots were produced to visualize the associations. The mean error is given by:

$$ME\begin{matrix} =\begin{matrix} \frac{1}{n} \end{matrix} \end{matrix}\sum_{i=1}^{n} (predicted\begin{matrix} -\begin{matrix} observed \end{matrix} \end{matrix})$$

## 5.3 Validation Results

Error: Reference source not found shows some of the validation results from the *P. falciparum* model and the *P. vivax* model. Model comparison via DIC, SES and DSS showed that M4 was better compared to other three models for both malaria species. The lower the predictive score the better the model. We calculated the mean error and the correlation using the 10% hold out set for the final model selects. The mean error was -0.442 and -0.306 for *P. falciparum* and *P. vivax* respectively. This showed and an overall tendency to underestimate both species. The correlation coefficient for *P. falciparum* was 0.62 and 0.63 for *P. vivax*.

Table 5.5: Model scoring parameters

|  | **Model** | **DIC** | **P_D_** | **Mlik (Integration)** | **Variance of predictive distribution** | **Std error of predictive distribution** | **ME** | **R^2^** | **SES** | **DSS** |
| --- | --- | --- | --- | --- | --- | --- | --- | --- | --- | --- |
| *P. falciparum* | M1 | 3670.00 | 86.80 | -1824.57 | 0.002 | 1.026 | - | - | 0.161 | 0.121 |
|  | M2 | 3596.90 | 95.60 | -1824.94 | 0.005 | 1.042 | - | - | 0.143 | 0.106 |
|  | M3 | 3599.48 | 90.64 | -1821.78 | 0.002 | 1.026 | - | - | 0.124 | 0.085 |
|  | M4 | 3570.76 | 96.85 | -1804.94 | 0.002 | 1.022 | -0.442 | 0.619 | 0.124 | 0.085 |
| *P. vivax* | M1 | 20933.49 | 203.48 | -10571.74 | 0.001 | 1.054 | - | - | 0.170 | 0.130 |
|  | M2 | 20781.31 | 301.97 | -10538.10 | 0.001 | 1.049 | - | - | 0.165 | 0.125 |
|  | M3 | 20935.46 | 206.49 | -10593.93 | 0.001 | 1.052 | - | - | 0.166 | 0.126 |
|  | M4 | 20780.64 | 301.46 | -10554.87 | 0.001 | 1.047 | -0.308 | 0.629 | 0.141 | 0.103 |

**References**

1. Department of Health (2009) World class commissioning for GP services: Improving GP access and responsiveness. In: Health Do, editor. Leeds, UK.

2. Laudicella M, Siciliani L, Cookson R (2012) Waiting times and socioeconomic status: evidence from England. Soc Sci Med 74: 1331-1341.

3. Singh P, Singh L, Kumar C, Rai R (2013) Correlates of maternal healthcare service utilisation among adolescent women in Mali: analysis of a nationally representative cross-sectional survey, 2006. Journal of Public Health 21: 15-27.

4. Jacobs B, Ir P, Bigdeli M, Annear PL, Van Damme W (2012) Addressing access barriers to health services: an analytical framework for selecting appropriate interventions in low-income Asian countries. Health Policy and Planning 27: 288-300.

5. The World Bank (2012) Harmonized list of fragile situations FY13. Fragile, conflict and violence.

6. Ameli O, Newbrander W (2008) Contracting for health services: effects of utilization and quality on the costs of the Basic Package of Health Services in Afghanistan. Bull World Health Organ 86: 920-928.

7. Apparicio P, Seguin A-M (2006) Measuring the Accessibility of Services and Facilities for Residents of Public Housing in Montreal. Urban Studies 43: 187-211.

8. Guagliardo MF (2004) Spatial accessibility of primary care: concepts, methods and challenges. Int J Health Geogr 3: 3.

9. Cromley EK, McLafferty SL (2010) GIS and Public Health. New York: Guilford Press.

10. Schoeps A, Gabrysch S, Niamba L, Sié A, Becher H (2011) The Effect of Distance to Health-Care Facilities on Childhood Mortality in Rural Burkina Faso. American Journal of Epidemiology 173: 492-498.

11. Comber A, Brunsdon C, Radburn R (2011) A spatial analysis of variations in health access: linking geography, socio-economic status and access perceptions. International Journal of Health Geographics 10: 44.

12. Noor AM, Amin AA, Gething PW, Atkinson PM, Hay SI, et al. (2006) Modelling distances travelled to government health services in Kenya. Trop Med Int Health 11: 188-196.

13. Gething PW, Noor AM, Zurovac D, Atkinson PM, Hay SI, et al. (2004) Empirical modelling of government health service use by children with fevers in Kenya. Acta Trop 91: 227-237.

14. Tanser F, Gijsbertsen B, Herbst K (2006) Modelling and understanding primary health care accessibility and utilization in rural South Africa: an exploration using a geographical information system. Soc Sci Med 63: 691-705.

15. Tanser F, Hosegood V, Benzler J, Solarsh G (2001) New approaches to spatially analyse primary health care usage patterns in rural South Africa. Trop Med Int Health 6: 826-838.

16. Tanser F, le Sueur D (2002) The application of geographical information systems to important public health problems in Africa. International Journal of Health Geographics 1: 4.

17. De Vries JJ, Nijkamp P, Rietveld P (2009) Exponential or power distance-decay for commuting? An alternative specification. Environment and Planning A 41: 461-480.

18. Wang L (2007) Immigration, ethnicity, and accessibility to culturally diverse family physicians. Health Place 13: 656-671.

19. Thill J-C, Kim M (2005) Trip making, induced travel demand, and accessibility. Journal of Geographical Systems 7: 229-248.

20. Blanford J, Kumar S, Luo W, MacEachren A (2012) It's a long, long walk: accessibility to hospitals, maternity and integrated health centers in Niger. International Journal of Health Geographics 11: 24.

21. Iyalomhe GB, Iyalomhe SI (2012) Health-seeking behavior of rural dwellers in southern Nigeria: Implications for healthcare professionals. International Journal of Tropical Disease & Health 2: 62-71.

22. Alegana V, Wright J, Petrina U, Noor A, Snow R, et al. (2012) Spatial modelling of healthcare utilisation for treatment of fever in Namibia. International Journal of Health Geographics 11: 6.

23. Luo W (2004) Using a GIS-based floating catchment method to assess areas with shortage of physicians. Health Place 10: 1-11.

24. Okiro E, Alegana V, Noor A, Mutheu J, Juma E, et al. (2009) Malaria paediatric hospitalization between 1999 and 2008 across Kenya. BMC Medicine 7: 75.

25. NoorAli R, Luby S, Rahbar MH (1999) Does use of a government service depend on distance from the health facility? Health Policy Plan 14: 191-197.

26. Buor D (2004) Gender and the utilisation of health services in the Ashanti Region, Ghana. Health Policy 69: 375-388.

27. Fortney J, Rost K, Warren J (2000) Comparing Alternative Methods of Measuring Geographic Access to Health Services. Health Services and Outcomes Research Methodology 1: 173-184.

28. Müller I, Smith T, Mellor S, Rare L, Genton B (1998) The effect of distance from home on attendance at a small rural health centre in Papua New Guinea. International Journal of Epidemiology 27: 878-884.

29. Huerta Munoz U, Kallestal C (2012) Geographical accessibility and spatial coverage modeling of the primary health care network in the Western Province of Rwanda. International Journal of Health Geographics 11: 40.

30. Delamater P, Messina J, Shortridge A, Grady S (2012) Measuring geographic access to health care: raster and network-based methods. International Journal of Health Geographics 11: 15.

31. Bissonnette L, Wilson K, Bell S, Shah T (2012) Access to primary health care: Does neighbourhood of residence matter? Health &amp; Place.

32. Gething PW, Noor AM, Gikandi PW, Ogara EA, Hay SI, et al. (2006) Improving imperfect data from health management information systems in Africa using space-time geostatistics. PLoS Med 3: e271.

33. Cibulskis RE, Bell D, Christophel EM, Hii J, Delacollette C, et al. (2007) Estimating trends in the burden of malaria at country level. Am J Trop Med Hyg 77: 133-137.

34. Mueller I, Slutsker L, Tanner M (2011) Estimating the Burden of Malaria: The Need for Improved Surveillance. PLoS Med 8: e1001144.

35. Gething P, Van Boeckel T, Smith D, Guerra C, Patil A, et al. (2011) Modelling the global constraints of temperature on transmission of Plasmodium falciparum and P. vivax. Parasites & Vectors 4: 92.

36. Guerra CA, Gikandi PW, Tatem AJ, Noor AM, Smith DL, et al. (2008) The limits and intensity of Plasmodium falciparum transmission: implications for malaria control and elimination worldwide. PLoS Med 5: e38.

37. Guerra CA, Howes RE, Patil AP, Gething PW, Van Boeckel TP, et al. (2010) The International Limits and Population at Risk of *Plasmodium vivax* Transmission in 2009. PLoS Negl Trop Dis 4: e774.

38. Safi N, Leslie T, Rowland M (2009) Progress and Challenges to Malaria Control in Afghanistan. Afghanistan Annual Malaria Journal: 15-29.

39. Hijmans R, Cameron S, Parra J, Jones P, Jarvis A (2005) WORLDCLIM - a set of global climate layers (climate grids).

40. Scharlemann JrPW, Benz D, Hay SI, Purse BV, Tatem AJ, et al. (2008) Global Data for Ecology and Epidemiology: A Novel Algorithm for Temporal Fourier Processing MODIS Data. PLoS One 3: e1408.

41. Hay SI, Tatem AJ, Graham AJ, Goetz SJ, Rogers DJ, et al. (2006) Global Environmental Data for Mapping Infectious Disease Distribution. Advances in Parasitology: Academic Press. pp. 37-77.

42. NASA (2011) TRMM. Goddard, Maryland, USA: NASA Goddard Space Flight Center.

43. Huffman GJ, Bolvin DT (2011) TRMM and Other Data Precipitation Data Set Documentation. Goddard, Maryland, United States: Laboratory for Atmospheres, NASA Goddard Space Flight Center and Science Systems and Applications, Inc.

44. Huffman GJ (1997) Estimates of root-mean-square random error contained in finite sets of estimated precipitation. . J Appl Meteor 36: 191-1201.

45. Noor AM, Kinyoki DK, Mundia CW, Kabaria CW, Mutua JW, et al. (2014) The changing risk of Plasmodium falciparum malaria infection in Africa: 2000 - 10: a spatial and temporal analysis of transmission intensity. The Lancet.

46. Alegana VA, Atkinson PM, Wright JA, Kamwi R, Uusiku P, et al. (2013) Estimation of malaria incidence in northern Namibia in 2009 using Bayesian conditional-autoregressive spatial-temporal models. Spatial and Spatio-temporal Epidemiology 7: 25-36.

47. Ray N, Ebener S (2008) AccessMod 3.0: computing geographic coverage and accessibility to health care services using anisotropic movement of patients. International Journal of Health Geographics 7: 63.

48. Pinheiro J, Bates D (2002) Mixed Effects Models in S and S-Plus: Springer.

49. R Development Core Team (2010) R : A language and environment for statistical computing. Vienna, Austria: R foundation for statistical computing.

50. Tobler W (1993) Three presentations on geographical analysis and modeling: National Center for Geographic Information and Analysis. Santa Barbara, CA93106-4060: University of California, Santa Barbara.

51. Walter Z (2008) Bicycle speed and power calculation.

52. Ministry of Public Health (2008) Afghanistan Health Survey 2006: Estimates of Priority Indicators for Rural Afghanistan. Kabul: Johns Hopkins University/Indian Institute of Health Management Research and Islamic Republic of Afghanistan, Ministry of Public Health.

53. Acerra J, Iskyan K, Qureshi Z, Sharma R (2009) Rebuilding the health care system in Afghanistan: an overview of primary care and emergency services. International Journal of Emergency Medicine 2: 77-82.

54. Mashal T, Takano T, Nakamura K, Kizuki M, Hemat S, et al. (2008) Factors associated with the health and nutritional status of children under 5 years of age in Afghanistan: family behaviour related to women and past experience of war-related hardships. BMC Public Health 8: 301.

55. Belay T (2010) Building on Early Gains in Afghanistan’s Health, Nutrition and Population Sector: *Challenges and Options*. Washington (D.C.): World Bank.

56. Trani J-F, Bakhshi P, Noor AA, Lopez D, Mashkoor A (2010) Poverty, vulnerability, and provision of healthcare in Afghanistan. Social Science & Medicine 70: 1745-1755.

57. Trani JF, Barbou-des-Courieres C (2012) Measuring equity in disability and healthcare utilization in Afghanistan. Med Confl Surviv 28: 219-246.

58. Akin JS, Hutchinson P (1999) Health-care facility choice and the phenomenon of bypassing. Health Policy Plan 14: 135-151.

59. Leonard K, Mliga GR, Mariam DH (2002) Bypassing health centers in Tanzania: Revealed preferences for observable and unobservable quality. Columbia University, Department of Economics.

60. Joseph AE, Phillips DR (1984) Accessibility and utilization: Geographical perspectives on health care delivery. London, UK: Harper & Row.

61. Gething P, Atkinson P, Noor A, Gikandi P, Hay S, et al. (2007) A local space-time kriging approach applied to a national outpatient malaria dataset. Comput Geosci 33: 1337-1350.

62. Das A, Gupta RD, Friedman J, Pradhan M, Mohapatra C, et al. (2013) Community perceptions on malaria and care-seeking practices in endemic Indian settings: policy implications for the malaria control programme. Malaria Journal 12: 39.

63. Youssef RM, Alegana VA, Amran J, Noor AM, Snow RW (2010) Fever prevalence and management among three rural communities in the North West Zone, Somalia. East Mediterr Health J 16: 595-601.

64. Elmardi KA, Noor AM, Githinji S, Abdelgadir TM, Malik EM, et al. (2011) Self-reported fever, treatment actions and malaria infection prevalence in the northern states of Sudan. Malar J 10: 128.

65. Rue H, Held L (2005) Gaussian Markov Random Fields: Theory and Applications (Chapman & Hall/CRC Monographs on Statistics & Applied Probability): Chapman and Hall/CRC.

66. Rue Hv, Martino S (2007) Approximate Bayesian inference for hierarchical Gaussian Markov random field models. Journal of Statistical Planning and Inference 137: 3177-3192.

67. Rue H, Martino S, Chopin N (2009) Approximate Bayesian inference for latent Gaussian models by using integrated nested Laplace approximations. Journal of the Royal Statistical Society: Series B (Statistical Methodology) 71: 319-392.

68. Fahrmeir L, Lang S (2001) Bayesian inference for generalized additive mixed models based on Markov random field priors Journal of the Royal Statistical Society, Series C 2: 201-220.

69. Kloek T, Dijk HKv (1978) Bayesian Estimates of Equation System Parameters: An Application of Integration by Monte Carlo. Econometrica 46: 1-19.

70. Schrödle B, Held L (2010) A primer on disease mapping and ecological regression using INLA. Computational Statistics 26: 241-258.

71. Sahu SK, Bakar KS (2012) Hierarchical Bayesian autoregressive models for large space–time data with applications to ozone concentration modelling. Applied Stochastic Models in Business and Industry 28: 395-415.

72. Bernardinelli L, Pascutto C, Best NG, Gilks WR (1997) Disease mapping with errors in covariates. Stat Med 16: 741-752.

73. Martins T, Simpson D, lindgren F, Rue H (2013) Bayesian computing with INLA: new features. Trondheim, Norway: Department of Mathematical Sciences, Norwegian University of Science and Technology. 29 p.

74. Gneiting T, Raftery AE (2007) Strictly Proper Scoring Rules, Prediction, and Estimation. Journal of the American Statistical Association 102: 359-378.

75. Czado C, Gneiting T, Held L (2009) Predictive Model Assessment for Count Data. Biometrics 65: 1254-1261.

76. Czado C, Gneiting T, Held L (2009) Predictive model assessment for count data. Biometrics 65: 1254-1261.

77. Spiegelhalter DJ, Best NG, Carlin BP, Van Der Linde A (2002) Bayesian measures of model complexity and fit. Journal of the Royal Statistical Society: Series B (Statistical Methodology) 64: 583-639.
